# Conserved molecular chaperone PrsA stimulates protective immunity against group A *Streptococcus*

**DOI:** 10.1038/s41541-024-00839-7

**Published:** 2024-02-26

**Authors:** Chien-Yu Lai, Jia-Xun Xie, Meng-Chih Lai, Zhao-Yi Wu, Jr-Shiuan Lin, Yu-Tsung Huang, Chia-Yu Chi, Chuan Chiang-Ni, Mark J. Walker, Yung-Chi Chang

**Affiliations:** 1 Graduate Institute of Microbiology, College of Medicine, National Taiwan University, Taipei, 10051 Taiwan; 2 Graduate Institute of Immunology, College of Medicine, National Taiwan University, Taipei, 10051 Taiwan; 3 Department of Laboratory Medicine, National Taiwan University Hospital, College of Medicine, National Taiwan University, Taipei, 10051 Taiwan; 4 National Institute of Infectious Disease and Vaccinology, National Health Research Institutes, Miaoli, 300 Taiwan; 5 Department of Microbiology and Immunology, College of Medicine, Chang Gung University, Taoyuan, 333 Taiwan; 6 Centre for Superbug Solutions, Institute for Molecular Bioscience, The University of Queensland, Brisbane, QLD Australia

**Keywords:** Protein vaccines, Bacterial infection

## Abstract

Group A *Streptococcus* (GAS) is a significant human pathogen that poses a global health concern. However, the development of a GAS vaccine has been challenging due to the multitude of diverse M-types and the risk of triggering cross-reactive immune responses. Our previous research has identified a critical role of PrsA1 and PrsA2, surface post-translational molecular chaperone proteins, in maintaining GAS proteome homeostasis and virulence traits. In this study, we aimed to further explore the potential of PrsA1 and PrsA2 as vaccine candidates for preventing GAS infection. We found that PrsA1 and PrsA2 are highly conserved among GAS isolates, demonstrating minimal amino acid variation. Antibodies specifically targeting PrsA1/A2 showed no cross-reactivity with human heart proteins and effectively enhanced neutrophil opsonophagocytic killing of various GAS serotypes. Additionally, passive transfer of PrsA1/A2-specific antibodies conferred protective immunity in infected mice. Compared to alum, immunization with CFA-adjuvanted PrsA1/A2 induced higher levels of Th1-associated IgG isotypes and complement activation and provided approximately 70% protection against invasive GAS challenge. These findings highlight the potential of PrsA1 and PrsA2 as universal vaccine candidates for the development of an effective GAS vaccine.

## Introduction

*Streptococcus pyogenes*, also known as group A *Streptococcus* (GAS), is a Gram-positive human-specific pathogen. Infections caused by GAS range from pharyngitis and impetigo to more severe conditions including bacteremia, puerperal sepsis, necrotizing fasciitis, scarlet fever, streptococcal toxic shock syndrome, and autoimmune complications such as acute rheumatic fever (ARF), rheumatic heart disease (RHD), and acute post-streptococcal glomerulonephritis (PSGN)^1,2^. Annually, GAS causes over 500,000 deaths and 18 million people are estimated to suffer from severe GAS-associated diseases^3,4^. GAS infections also contribute significantly to antibiotic misuse, with sore throat (pharyngitis) being the third most common condition leading to antibiotic prescriptions in the U.S. and is a primary reason for self-medication with antibiotics in Europe^5–8^. These observations highlight the urgent need for the development of a GAS vaccine. A safe and efficacious vaccine would not only help control GAS infections and mitigate associated complications but also contribute to a reduction of antibiotic misuse^9^.

Vaccine development for GAS has primarily focused on the major cell surface M protein, encoded by the *emm* gene. Although M protein is highly immunogenic, it exhibits high genetic diversity, with more than 200 *emm*-types identified, showing remarkable variation in geographic distribution and disease spectrum^10^. The development of a safe vaccine is further complicated by the potential cross-reactivity between host-generated antibodies against the M protein and human proteins, which could possibly lead to autoimmune diseases^11,12^. To address these concerns and increase vaccine coverage, there is growing interest in exploring conserved non-M protein antigens. Several surface-anchored and secreted virulence factors have been identified as potential non-M protein candidates for vaccine targets, and immunization with these proteins elicits protective efficacy in preclinical models^13–17^.

In our previous research, we identified the GAS lipoproteins PrsA1 and PrsA2 as key players in the export and maturation of numerous virulence factors^18^. A large-scale genomic study encompassing 2083 genetically diverse GAS strains revealed that both *prsA1* and *prsA2* belong to the core genome, indicating high prevalence and conservation across the genetically diverse GAS population^19^. In this study, we investigate whether PrsA could serve as a viable vaccine candidate capable of eliciting broad protection across diverse GAS serotypes. We found that antibodies targeting PrsA1 and PrsA2, which do not cross-react to human heart tissues, exhibit bactericidal activity against multiple GAS serotypes in vitro and conferred protective immunity when passively transferred to infected mice. Furthermore, when formulated with the Th1-promoting adjuvant CFA, PrsA1 and PrsA2 elicited a protective response against systemic GAS infection in a mouse intraperitoneal challenge model. These results suggest that PrsA is a promising M-type-independent vaccine candidate to prevent GAS infections.

## Results

### PrsA is highly conserved among GAS strains, immunogenic, and does not induce antibodies recognizing human heart proteins

**Fig. 1:**
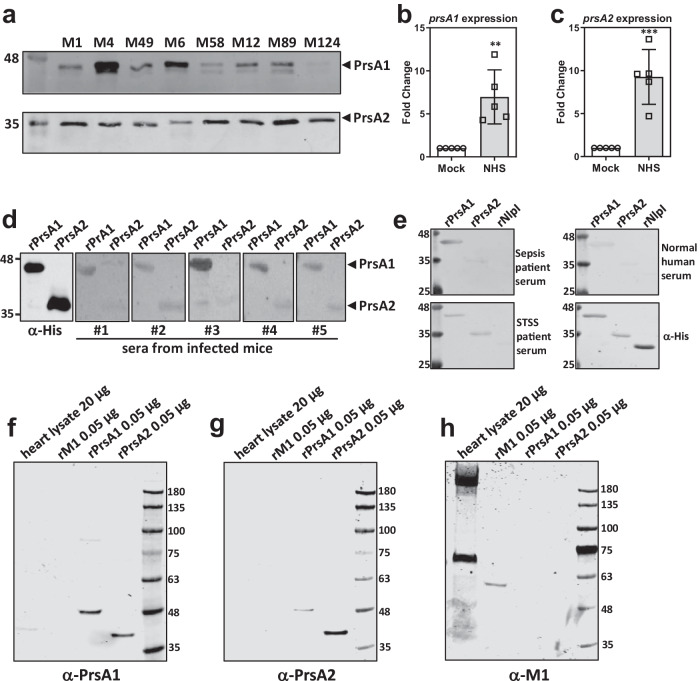
Serotype distribution and immunogenicity of PrsA1 and PrsA2. **a** Western blot detection of PrsA1 and PrsA2 in crude membrane extracts collected from eight representative clinical GAS isolates of different *emm*-types. (M1 strain A20, M4 strain 6043-05, M49 strain NTU43, M6 strain NTU45, M58 strain NTU46, M12 strain NTU25, M89 strain NTU30, M124 strain NTU32). **b**, **c** Expression of *prsA1* and *prsA2* in response to human serum. M1 GAS (strain A20) were treated with RPMI 1640 medium (Mock) or RPMI 1640 medium with 10% normal human serum (NHS) for 30 min, followed by analyzing the transcript levels of *prsA1* (**b**) and *prsA2* (**c**) by RT-qPCR analysis. Data shown were normalized with *recA*, *gyrsA* and *proS* and presented as means ± SD from five independent experiments. Statistical analysis was performed using Student’s *t* test. ** <0.01; *** <0.005. **d** His-tagged PrsA1 and PrsA2 proteins (0.5 µg) were separated by 10% SDS-PAGE and probed with murine sera collected from GAS-infected mice (1:500 dilution). **e** His-tagged PrsA1, PrsA2 and NlpI proteins (0.5 µg) were separated by 10% SDS-PAGE and probed with human sera obtained from patients with invasive GAS infections and healthy adults (1:300 dilution). **f**–**h** Serum IgG cross-reactivity to human heart tissue proteins. Western blot analysis of human heart lysates with antisera from rabbits immunized with PrsA1 (**f**) and PrsA2 (**g**) or mice immunized with M1 proteins (**h**). Purified recombinant PrsA1, PrsA2 and M1 proteins were included as positive controls.

An ideal vaccine antigen for Group A *Streptococcus* (GAS) should possess high prevalence and sequence conservation across the genetically diverse GAS population. Previous studies have indicated that both *prsA1* (M5005_Spy1133) and *prsA2* (M5005_Spy1732) genes are highly prevalent, present in more than 99% of >2000 GAS genomes^19^. To further investigate the amino acid sequence variation of PrsA1 and PrsA2, we analyzed 264 published GAS genomes with diverse *emm* and ST types. Our analysis revealed that both PrsA1 and PrsA2 exhibit low levels of naturally occurring sequence variation, with more than 98% sequence identity observed across the examined strains (Supplementary Fig. 1a, b). In addition, both PrsA1 and PrsA2 can be detected across multiple GAS isolates from patients with different forms of disease, albeit with varying expression levels (Fig. 1a).

GAS is known to swiftly adapt to the host environment and regulate multiple virulence-related genes^20,21^. We further investigated whether the expression of *prsA1* and *prsA2* is regulated during the course of infection. To simulate the host environment, GAS was incubated with pooled normal human serum (NHS). Compared to untreated GAS (Mock), both *prsA1* (Fig. 1b) and *prsA2* (Fig. 1c) were significantly upregulated in NHS-treated GAS, suggesting responsiveness to the host environment. Furthermore, we found that mice that had previously survived GAS infections, but not uninfected mice, developed antibodies recognizing PrsA1 and PrsA2, suggesting that GAS-infected mice generated specific antibodies against PrsA1 and PrsA2 (Fig. 1d and Supplementary Fig. 2a). In addition, human sera obtained from patients with invasive GAS infections exhibited strong reactivity to PrsA1 and PrsA2. Although both PrsA1 and PrsA2 were poorly recognized by normal human serum, PrsA1 exhibited a stronger signal compared to PrsA2 (Fig. 1e). These findings collectively confirm the expression and immune recognition of PrsA1 and PrA2 during infection, with PrsA1 showing slightly superior immune recognition compared to PrsA2.

Ensuring the safety of streptococcal vaccines is crucial, particularly in avoiding immune responses that cross-react with self-antigens in heart tissues, that may contribute to the pathogenesis of rheumatic heart disease (RHD). To evaluate the potential for autoimmunity, we conducted western blot analysis to examine the cross-reactivity of PrsA1- and PrsA2-specific antibodies to host heart proteins. Notably, the M1 antisera showed significant cross-reactivity to human heart tissues, targeting high molecular weight components of the heart lysates (Fig. 1h). In contrast, minimal, if any, cross-reactivity to human heart tissues was observed for the PrsA1- and PrsA2-specific antibodies purified from rabbit antisera (Fig. 1f, g) and antisera collected from PrsA immunized mice (Supplementary Fig. 2b, c). Furthermore, we examined the histology of hearts from mice receiving twice PrsA immunization and found no observable differences in necrosis and inflammatory infiltrates compared to that of OVA-immunized mice (Supplementary Fig. 2d). These findings suggest that antibodies against PrsA1 and PrsA2 exhibit limited cross-reactivity to host heart proteins, supporting their potential as safe vaccine antigens.

### PrsA-specific antibodies bind to multiple GAS serotypes and demonstrate functional killing activity

**Fig. 2:**
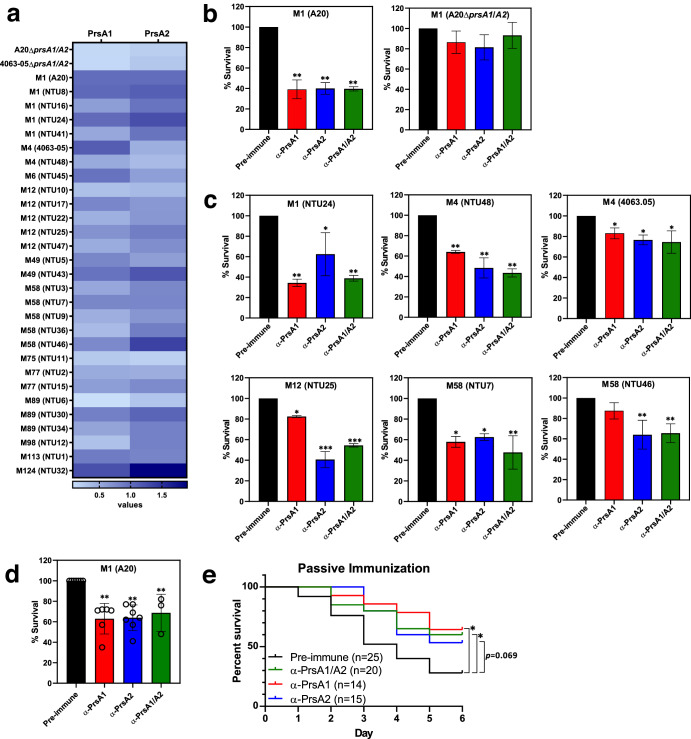
Evaluation of rabbit PrsA antisera in GAS surface binding, opsonophagocytic killing and passive protection in mice. **a** Twenty-nine GAS strains of different *emm*-types were used to determine the bacterial surface targeting ability of PrsA-specific antibodies. The heat map values were calculated by subtracting the reads of PrsA-specific antibody group from the reads of the control IgG group and normalized to the values obtained from M1 A20 strain. Opsonophagocytic killing of GAS M1 A20 and its isogenic *prsA1/A2* deficient mutant (**b**) and clinical GAS isolates with different *emm*-types (**c**) by human neutrophils in the presence of PrsA1 antisera, PrsA2 antisera or in combination compared to pre-immune sera. Data presented here were combined, normalized and expressed as means ± SD from two independent experiments, each performed in biological triplicates. **d** Human whole blood killing assay. M1 GAS (strain A20) was grown in human whole blood with the addition of rabbit control antibodies, purified rabbit anti-PrsA1 antibodies, anti-PrsA2 antibodies or in combination. Percent survival of GAS was calculated in comparison to the number of GAS surviving in the control antibody group. Each dot in the figure represents the corresponding value in each experiment. Data presented here were combined, normalized and expressed as means ± SD. **e** Kaplan–Meier survival curve of mice. Groups of female ICR mice (*n* = 15–25, pooled data from 2–3 independent experiments) were injected intraperitoneally with rabbit antisera raised against purified PrsA1 and PrsA2 or with pre-immune sera. Two hours later, the mice were challenged intraperitoneally with lethal dose of M1 GAS strain NTU24. A log rank test was used to compare the survival of PrsA antisera-treated animals with animal receiving pre-immune sera. One-way ANOVA with Tukey’s post hoc method was used for pairwise comparisons (**b**–**d**). * <0.05; ** <0.01; *** <0.005.

To investigate the presence and accessibility of PrsA1 and PrsA2 on the bacterial surface, intact clinical GAS isolates of different *emm*-types were analyzed using a whole bacteria ELISA assay with PrsA1- and PrsA2-specific rabbit polyclonal antibodies. As shown in Fig. 2a, PrsA1- and PrsA2-specific antibodies exhibited clear binding to WT M1 GAS A20 strain and M4 GAS 4063-05, while no binding was observed with the isogenic mutant lacking *prsA1* and *prsA2* (A20Δ*prsA1/A2* and 4063-05Δ*prsA1/A2*). Importantly, significant binding of PrsA1- and PrsA2-specific antibodies to GAS was observed in nearly all tested clinical GAS isolates, confirming that PrsA1 and PrsA2 are expressed and exposed on the bacterial surface.

To determine whether PrsA antisera could enhance bacterial clearance, neutrophil opsonophagocytic killing (OPK) assays were performed against different *emm*-type GAS strains. As shown in Fig. 2b, the presence of PrsA1 antisera, PrsA2 antisera, or a combination of both significantly enhanced neutrophil OPK function against the M1 A20 strain. However, this effect was not observed in the isogenic mutant lacking both *prsA1* and *prsA2* (A20Δ*prsA1*/*prsA2*). Furthermore, despite variations in the levels of PrsA1 and PrsA2 expression in clinical GAS isolates, enhanced neutrophil OPK activity was also evident in these isolates when PrsA1 antisera, PrsA2 antisera, or a combination of both were introduced (Fig. 2c). Additionally, in an ex vivo human whole blood bactericidal assay, the growth of GAS was notably reduced in the presence of anti-PrsA1 antibodies, anti-PrsA2 antibodies or a combination of both compared to control rabbit IgG antibody (Fig. 2d).

To assess the in vivo efficacy of PrsA antisera, rabbit PrsA1 and PrsA2 antisera, either individually or combined, were transferred into adult ICR mice 2 h before intraperitoneal lethal dose GAS challenge. Control mice receiving pre-immune rabbit sera showed less than 30% survival within 6 days of infection. In contrast, mice receiving PrsA1 antisera, PrsA2 antisera or the combination of both showed significant protection against mortality, with more than 60% survival over the course of the experiment (Fig. 2e). Additionally, mice that received PrsA antisera, heat-inactivated at 56 °C for 30 min, also demonstrated higher survival rates compared to those that received heat-inactivated pre-immune sera (Supplementary Fig. 2e). These findings confirm the specific role of PrsA1/A2 antisera in protecting mice against GAS infection. Collectively, our data demonstrate that PrsA1- and PrsA2-specific antibodies bind to the surface of GAS, enhance human neutrophil OPK function against GAS strains of various *emm*-types, and induce protective immunity in infected mice following passive transfer.

### Vaccine efficacy of PrsA

**Fig. 3:**
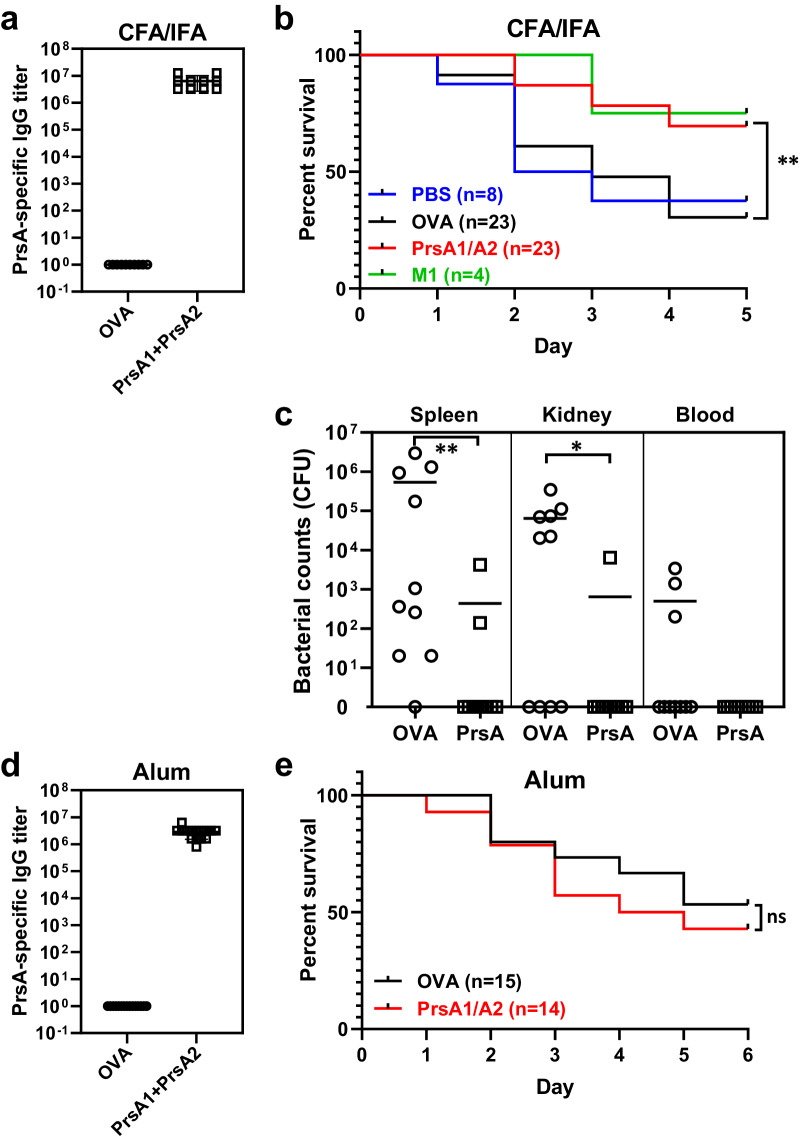
Immunization with PrsA1/A2 proteins protects against systemic GAS infection. **a**–**c** Female ICR mice were immunized i.m. with indicated CFA/IFA-adjuvanted antigens (OVA, PrsA1/A2 and M1 proteins) or PBS control. **a** Levels of PrsA1/A2-specific IgG in sera from mice 14 days after a second immunization were determined by ELISA. **b** Immunized mice were challenged intraperitoneally with lethal doses (5–6 × 10^7^) of M1 GAS strain NTU24 and monitored for survival. **c** Bacterial loads in spleen, kidney and blood were determined 18 h post-infection in mice infected with sublethal doses (2 × 10^7^) of GAS. **d**, **e** Female ICR mice were immunized s.c. with alum-adjuvanted PrsA1/A2. **d** Levels of PrsA1/A2-specific IgG in sera from mice 14 days after a second immunization were determined by ELISA. **e** Immunized mice were challenged intraperitoneally with lethal doses (5–6 × 10^7^) of M1 GAS strain NTU24 and monitored for survival. IgG titers were defined as the highest dilution of serum that gave an absorbance exceeded the mean absorbance of negative control plus 3 standard deviations. Each dot in the figure represents the corresponding value of each mouse. Mann–Whitney *U* test was used to compare the bacterial burden in tissues (**c**). A log rank test was used to compare the survival of animals (**b**, **e**). * <0.05; ** <0.01; ns not significant.

The conserved nature of PrsA within GAS strains, its immunogenicity, absence of human homologs, and surface exposure prompted us to investigate its potential as a protective vaccine antigen. PrsA1 and PrsA2 proteins were administered to ICR mice via intramuscular immunization with CFA/IFA or subcutaneous immunization with alum. Following immunization, high levels of serum IgG antibodies against PrsA1 and PrsA2 were detected, with geometric mean titers of 6.4×10^6^ for CFA/IFA immunization (Fig. 3a) and 1.6 × 10^6^ for alum (Fig. 3d). In contrast, OVA-immunized mice did not show measurable titers against PrsA1/A2. To assess the protective efficacy of PrsA1/A2 immunization against systemic infection, mice were intraperitoneally challenged with M1 GAS following the final immunization. As shown in Fig. 3b, PrsA/CFA/IFA immunization resulted in 69% survival rate, only slightly lower than that of M1-immunized mice (75% survival rate). In contrast, unimmunized PBS-injected mice and control OVA-immunized mice showed only 30% survival rate. Moreover, disseminated bacterial loads recovered from the spleens and kidneys of PrsA/CFA/IFA immunized mice were significantly lower than those of OVA controls, and none of the PrsA/CFA/IFA immunized animals developed bacteremia 18 h post intraperitoneal infection (Fig. 3c). In contrast to the protective immunity elicited by PrsA/CFA/IFA immunization, PrsA/alum immunization failed to confer protection, with a survival rate of 42%, comparable to the 53% survival rate observed in the OVA-immunized mice (Fig. 3e). These results suggest that, in addition to antibody titer, factors affecting antibody effector functions, such as complement fixation or Fcγ receptor (FcγR) engagement, may contribute to the variations in protection observed among PrsA-vaccinated animals.

### Characterization of IgG isotype profile and functional activity

**Fig. 4:**
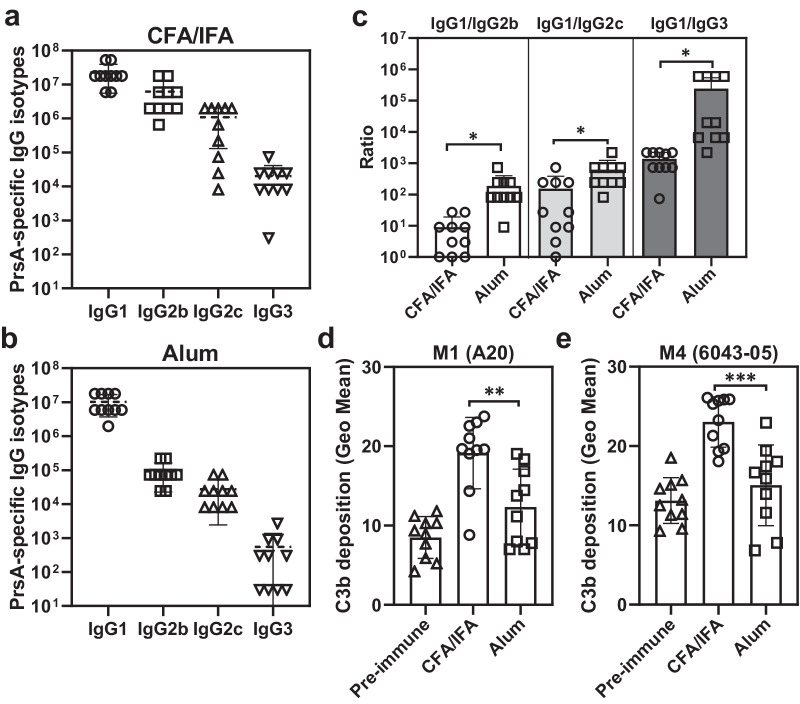
PrsA-specific IgG subclass profile and complement-fixation capability. The subclass profile of PrsA-specific serum IgG antibodies in PrsA-immunized mice adjuvanted with CFA/IFA (**a**) and alum (**b**). Serum titers of PrsA-specific IgG1, IgG2b, IgG2c and IgG3 were determined by ELISA using isotype specific secondary IgG. Individual IgG subclass titers were defined as the highest dilution of serum for which OD_450_ exceeded the mean OD_450_ of negative control plus 3 standard deviations. **c** The ratio of IgG1/IgG2b, IgG1/Ig2c and IgG1/IgG3. The data shown in (**c)** was extrapolated from (**a**, **b**) by dividing the titer of IgG1 by the titer of IgG2b, IgG2c and IgG3, respectively. **d**, **e** C3b deposition on GAS. M1 strain A20 (**d**) and M4 strain 6043-05 (**e**) were incubated with 1% vaccinated mouse sera for 30 min and the bacterial surface-deposited C3b was detected by flow cytometer with rabbit anti-complement C3 antibody. Each dot in the figure represents the corresponding value of each mouse. Bars represent means ± SD. Significance was determined by Mann–Whitney *U* test (**c**) or one-way ANOVA with Tukey’s post hoc test (**d**, **e**). * <0.05; ** <0.01; *** <0.005.

The choice of adjuvant plays a crucial role in shaping the antibody response and determining the subclass and level of antibodies generated. Despite both adjuvants eliciting comparable levels of PrsA1/A2-specific IgG1 antibodies, CFA/IFA immunization resulted in significantly higher levels of PrsA1/A2-specific IgG2b, IgG2c, and IgG3 antibodies compared to alum immunization (Fig. 4a, b). Furthermore, the ratios of IgG1/IgG2b, IgG1/IgG2c, and IgG1/IgG3, which provide an indication of the T helper (Th) 2/Th1 bias, were significantly lower in mice immunized with CFA/IFA, indicating a more pronounced Th1 response was generated in the CFA/IFA-vaccinated animals (Fig. 4c).

Different IgG subclasses exhibit varying affinity for complement and FcγR on immune cells, leading to different functional activities against microbes and tumors^22,23^. The major GAS virulence factor, hyaluronic acid (HA) polysaccharide capsule, has been shown to impede antibodies from targeting surface-exposed antigens and C3b deposition, thereby conferring resistance to phagocytosis^24,25^. Therefore, we analyzed the complement-fixation capacities of PrsA/alum sera and PrsA/CFA/IFA sera on both encapsulated M1 GAS A20 strain^26^ and nonencapsulated M4 GAS strain 4063-05 which lacks the entire *hasABC* operon required for HA capsule biosynthesis^27^. GAS was incubated with individual mouse sera obtained after booster immunization and stained for C3b deposition on the bacterial surface. As shown in Fig. 4d, e, sera from PrsA/CFA/IFA immunized mice induced significantly higher levels of C3b deposition on both capsule-positive and capsule-negative GAS bacterial surfaces, compared to sera from PrsA/alum immunized mice. This observation is consistent with the higher levels of IgG2b, IgG2c, and IgG3 antibodies in CFA/IFA-vaccinated mice.

### Vaccination-induced CD4^+^ T cell responses

**Fig. 5:**
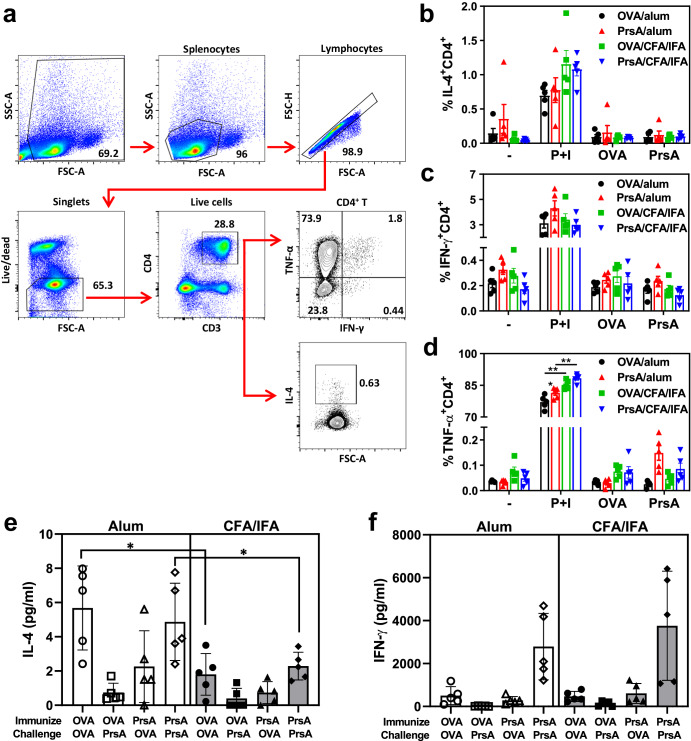
Antigen-specific T cell responses. ICR mice were immunized on days 0 and 21 with OVA/alum, PrsA/alum, OVA/CFA/IFA or PrsA/CFA/IFA. Spleens were harvest 1 week post last immunization. Total splenocytes (10^6^) were stimulated with media alone (−), PMA (20 ng/ml) plus ionomycin (1 µg/ml) (P + I), OVA or PrsA proteins (5 µg/ml) for 6 h, and cytokine (IL-4, IFN-γ and TNF-α) expression in CD4 T cells was evaluated by intracellular cytokine stain. **a** Flow cytometry contour plots representing the gating strategy used to identify IL-4^+^CD4^+^, IFN-γ^+^CD4^+^, TNF-α^+^CD4^+^ T cells after P + I stimulation. The frequency of IL-4^+^CD4^+^ (**b**), IFN-γ^+^CD4^+^ (**c**), TNF-α^+^CD4^+^ (**d**) T cells in each treatment group. **e**, **f** Antigen-specific cytokine production. Total splenocytes (10^6^) from mice immunized with OVA/Alum, PrsA/alum, OVA/CFA/IFA or PrsA/CFA/IFA were stimulated with OVA or PrsA proteins (1 µg/ml) for 72 h. The accumulated IL-4 and IFN-γ released to the culture supernatants were analyzed by ELISA. Each dot in the figure represents the corresponding value of each mouse. Bars represent means ± SD. Significance was determined by two-way ANOVA with Tukey’s multiple comparison post hoc test (**b**–**d**) or Mann–Whitney *U* test (**e**, **f**). * <0.05; ** <0.01; *** <0.005.

CFA and alum have been shown to preferentially induce Th1 and Th2 responses, respectively^28,29^. In mice, the Th2-prone response is associated with the production of IgG1 antibodies, while a Th1 response is linked with the generation of IgG2a, IgG2b, and IgG3 antibodies. To analyze the production of Th1 (IFN-γ and TNF-α) and Th2 (IL-4) cytokines in vaccine-induced antigen-specific T cells, we conducted intracellular cytokine staining (ICS, Fig. 5a) on immunized splenocytes from immunized mice after in vitro stimulation with OVA, PrsA or a general activator, PMA (phorbol 12-myristate 13-acetate) and ionomycin (P + I). Despite CD4^+^ T cells from immunized mice showing barely detectable levels of IL-4, IFN-γ and TNF-α production in response to corresponding antigen stimulation, they exhibited a robust response to P + I stimulation (Fig. 5b–d and Supplementary Fig. 5). This observation suggests that the antigen-specific T cells may exist in a very low frequency in the immunized ICR mice, possibly due to the genetic background of outbred ICR mice, making it challenging to detect their responses using the ICS technique. Nevertheless, the robust response to P + I stimulation suggests that the T cells in the immunized mice are functional and can be activated. Notably, the percentage of TNF-α secreting CD4^+^ T cells were slightly but significantly higher in the CFA/IFA-adjuvanted group upon P + I stimulation compared to alum-adjuvanted group (Fig. 5d), implying a stronger Th1 response in CFA/IFA-adjuvanted mice. Subsequently, to test whether antigen-specific cytokine production could accumulate and be detected, we measured the IL-4 and IFN-γ secreted into the culture supernatant of stimulated splenocytes after 72 h. As shown in Fig. 5e, PrsA/alum-vaccinated splenocytes stimulated with PrsA protein induced significantly higher IL-4 secretion compared to PrsA/CFA/IFA-vaccinated splenocytes. In addition, although it did not reach statistical significance, there was a trend indicating higher IFN-γ secretion in splenocytes from CFA/IFA-adjuvanted mice upon PrsA protein stimulation compared to alum-adjuvanted mice (Fig. 5f). These results highlight that the choice of adjuvant used for PrsA immunization significantly impacts both T cell responses and overall magnitude and functional activity of the antibody response, which likely contributes to the differences in protection observed among PrsA-vaccinated animals.

## Discussion

In this study, we examined the immunogenicity and protective efficacy of PrsA, a molecular chaperone, against GAS infection. Our findings demonstrate that PrsA1 and PrsA2 are upregulated in conditions that mimic the host, highly immunogenic and conserved among GAS strains. Importantly, antibodies targeting PrsA1 and PrsA2, which did not cross-react to human heart tissues, showed bactericidal activity against multiple GAS strains in vitro and conferred protective immunity when passively transferred to infected mice. Furthermore, when formulated with the Th1-promoting adjuvant CFA, PrsA1 and PrsA2 elicited a protective response against systemic GAS infection in a mouse intraperitoneal challenge model. These results suggest that PrsA holds promise as a universal vaccine candidate for effective protection against GAS infection.

Developing an effective GAS vaccine requires achieving broad coverage while avoiding cross-reactivity with host tissues. Previous studies have shown that antibodies generated by M protein vaccination, although highly immunogenic, cross-react with human proteins, raising safety concerns and hindering the development of M-protein-based vaccines^11,12^. GAS exhibits extensive genetic diversity, with over 200 *emm*-types identified, resulting in significant variation in geographic distribution and disease spectrum^10^. Hence, there is growing interest in exploring conserved non-M protein candidates to enhance serotype coverage and prevent the generation of cross-reactive antibodies associated with RHD^13–15,30,31^. Our study, along with others, demonstrated the prevalence and conservation of *prsA1* and *prsA2* across the genetically diverse GAS population^19^. Importantly, both PrsA1 and PrsA2 showed no identified similarity to human UniProtKB reference proteome when conducting a protein similarity search with NCBI BLAST+ with an expectation value of 0.0001, a setting where the M protein showed similarity to several human proteins. Moreover, antibodies targeting these proteins did not react with human heart extracts, in contrast to antibodies targeting the M1 protein (Fig. 1f–h), suggesting that PrsA is less likely to trigger autoimmune responses.

Prior studies have shown that PrsA1 and PrsA2 can be detected using human sera obtained from patients with pharyngitis, whereas such detection is absent in plasma from healthy adults^13,32,33^. Consistent with previous reports, we found that PrsA1 and PrsA2 were poorly recognized by normal human serum, while both proteins were positively recognized by sera obtained from patients with invasive GAS infections (Fig. 1e). In contrast to PrsA1 and PrsA2, antibody responses against two GAS conserved antigens, streptococcal C5a peptidase and immunogenic secreted protein, can still be observed in sera collected from both children and adults without confirmed GAS infection within the past 3 months^34^. These findings suggest that the expression and immunogenicity of individual GAS antigens, as well as the titer and duration of induced antibodies, may differ in individuals with recent invasive infection, benign infection or asymptomatic colonization.

One of the major GAS virulence factors, hyaluronic acid (HA) polysaccharide capsule, has been shown to play an important role in preventing phagocytic killing by impeding antibodies from targeting surface-exposed antigens, hindering C3b deposition, and preventing phagocytes from interacting with the C3b deposited on the bacterial cell surface^24,25,35,36^. The HA capsule is not universally present in all GAS strains, such as M4 and M22 serotypes, and some M89 serotypes lack the *hasABC* operon and are deficient in the expression of HA capsule. These observations suggest that different GAS strains may employ distinct strategies to evade phagocytic killing. Therefore, we tested the accessibility of surface PrsA and the complement activation capability of PrsA-specific antibody on both encapsulated and nonencapsulated GAS strains. Our results demonstrate that PrsA-specific antibody can recognize PrsA1 and PrsA2 in both encapsulated (such as M1 GAS) and nonencapsulated GAS isolates (such as M4 GAS) (Fig. 2a). Moreover, efficient C3b deposition was observed on both encapsulated and nonencapsulated GAS strains in the presence of PrsA/CFA/IFA antisera which exhibited higher levels of Th1-type IgGs (Fig. 4c–e). Together, our data suggest that PrsA-specific antibody can recognize PrsA and initiate C3 activation on GAS surface regardless of it capsule.

The presence of PrsA1 and PrsA2 antisera significantly enhanced the neutrophil OPK activity against various clinical GAS isolates (Fig. 2c). The expression level of PrsA2 is higher than PrsA1 in GAS M12 strain NTU25 and M58 strain NTU46 (Figs. 1a and 2a), correlating with superior neutrophil OPK activity in the presence of PrsA2 antisera compared to PrsA1 antisera. However, we observed comparable bactericidal activity in most cases, regardless of whether PrsA1 antisera, PrsA2 antisera, or a combination of both were included in the OPK assay, despite variations in the expression levels of PrsA1 and PrsA2 among different clinical GAS isolates. This may be attributed to the high amino acid sequence identity between PrsA1 and PrsA2 (65% identity), even with certain segments in the N- and C-terminal regions exhibiting continuous identical amino acids between the two proteins (Supplementary Fig. 3a). This strongly suggests that antibodies generated by PrsA1 immunization can cross-react with PrsA2, and vice versa. This hypothesis is supported by the western blot results (Fig. 1f, g), demonstrating that rabbit PrsA1 and PrsA2 antisera, raised by immunization with PrsA1 and PrsA2, respectively, exhibit cross-reactivity with the other protein. Considering the prevalence of both PrsA1 and PrsA2 in GAS and the observed cross-reactivity of antibody raised through PrsA1 and PrsA2 immunization, it may be challenging to completely exclude the influence of another PrsA isoform when immunizing animals with only one of the PrsA isoforms. Therefore, we chose to incorporate both PrsA1 and PrsA2 into our vaccine formulation for the following immunization experiments.

Alum, commonly used as primary adjuvant to test GAS vaccine candidates, was effective in generating opsonizing antibodies and providing in vivo protection in M protein-vaccinated mice^13,37–39^. However, in our study, using alum as an adjuvant with PrsA did not confer protection against systemic lethal challenge in mice, despite this formulation generating high titers of PrsA-specific antibodies. Intriguingly, switching from alum to CFA/IFA as the adjuvant significantly improved the protective efficacy of PrsA (Fig. 3). Compared to alum, CFA, a Th1-inducing adjuvant, elicited higher levels of Th1-type IgGs, superior complement activation capability, and favorable Th1/Th2 immune responses in our studies (Figs. 4c–e and 5e, f). Opsonophagocytic killing, the primary host defense mechanism known for controlling Gram-positive bacterial infection, is predominantly associated with the generation of Th1-type IgG2 and IgG3 antibodies^40–44^. Animal studies have also demonstrated the superiority of the murine Th1-type antibody isotype in opsonophagocytosis, complement activation, and antibody-dependent cellular cytotoxicity^45–47^. In humans, higher levels of IFN-γ and IgG3 have been correlated with reduced susceptibility to GAS infection^34^. The observed differential adjuvant effects in eliciting protective immunity align with several recent reports^15,16,48,49^, suggesting that Th1-associated immune responses may be crucial for the protective efficacy of non-M protein-based vaccines.

Different administration routes were used for PrsA/alum and PrsA/CFA/IFA immunization in our studies, complicating the conclusion that the observed differences were sorely attributed to the adjuvant variance in the immunization protocol. Despite intramuscular and subcutaneous administration being known to preferentially target distinct anatomical compartments, similar levels of antigen uptake, as well as comparable magnitude and quality of antigen-specific cellular and humoral responses, were generated over time^50,51^. In a recent report, alum was shown to trigger lower Th1-type IgG2a production compared to squalene-based emulsion adjuvants, MF59, AS02 and AS03, regardless of whether delivered intramuscularly or subcutaneously. While the alum vaccine administrated via the intramuscular route generated comparable or slightly higher IgG2a levels than those inoculated via the subcutaneous route, the levels of IL-4 and IFN-γ induced by the alum adjuvant were not affected by inoculation routes^52^. Although we can’t exclude the potential influence of immunization routes on our experiments, our results nevertheless demonstrate that a more balanced Th1/Th2 responses can be elicited by CFA/IFA compared to alum.

A successful vaccine comprises two main components: an antigen for generating pathogen-specific immune responses and an adjuvant for enhancing the magnitude and durability of the immune response. Duo to the high levels of surface abundance and immunogenicity, several current GAS vaccine candidates focus on M protein antigens^31^. The 30-valent M protein-based vaccine, covering major GAS serotypes prevalent in North America and Europe, has completed the Phase I clinical trial and demonstrated good immunogenicity and tolerability without clinical evidence of autoimmune reactions, with a theoretical global coverage of 48%^38,53^. Moreover, peptide vaccines derived from the conserved C-terminal region of M protein, including StrepInCor, J8 and P*17, have also shown protective immunity and a lack of autoimmune reaction in various mouse infection models^37,54–58^. Substantial preclinical progress has also been achieved in the non-M protein vaccines by incorporating multiple GAS virulence factors, such as SpyCEP, SLO, SCPA, SpyAD, ADI, TF, T antigen and group A carbohydrate (GAC), into vaccine formulations^13–15,59^.

Recently, novel adjuvant formulations and delivery methods have emerged to enhance vaccine efficacy. The utilization of liposomal adjuvant CAF^®^ 01^60^, self-assembled poly (methyl acrylate) polymer nanoparticles^61^, immunostimulatory 3D(6-acyl) PHAD glycolipid^62^, biopolymer particles derived from endotoxin-free *E. coli*^63^, and high-density microarray patch^64^ on J8 and P*17 peptide vaccines has demonstrated a significant enhancement in the magnitude and duration of mucosal immunity or a preference for driving Th1-prone immune responses following immunization. The incorporation of potent immune-stimulants like saponin QS21 and TLR4 agonist 3D-(6-acyl) PHAD^16^ or TLR9 agonist CpG^30^ in non-M protein vaccines can induce Th1- or Th17-related immune responses to promote bacterial clearance. In our study, CFA/IFA served as a proof-of concept adjuvant to assess the protective immunity elicited by PrsA immunization in mice. Due to its toxicity and lack of human compatibility, the adaption of recently developed adjuvant formulations should greatly expand the application of PrsA as promising GAS vaccine candidate, meeting efficacy and safety requirements for future GAS vaccine development.

*Streptococcus* species represent a diverse and significant group of human and agricultural pathogens, characterized by a dynamic and open pan-genome that enables them to adapt to changing environmental conditions^65–67^. Despite the inherent diversity within the *Streptococcus* genus, PrsA is found in several *Streptococcus* species, including GAS, *S. agalactiae*, *S. anginosus*, *S. dysgalactiae*, *S. equi*, *S. gordonii*, *S. mitis*, *S. mutans, S. pneumoniae*, *S. sanuguinis*, and *S*. *suis*^68–72^, with amino acid sequence identities ranging from 40 to 75% (Supplementary Fig. 3b, c). While it has been demonstrated that PrsA from *S. suis* (SsPrsA) exhibits dose-dependent cytotoxicity, leading to significant cell death in murine RAW264.7 macrophages and bEND3.0 brain microvascular endothelial cells at concentrations above 75 μg/ml^73,74^, it is important to note that GAS PrsA1 and PrsA2 did not induce detectable cytotoxicity in RAW264.7 cells, even when tested at the same concentration at which SsPrsA triggered ~80% cell death (Supplementary Fig. 3d). Furthermore, the amino acid comparison demonstrated that SsPrsA shares low sequence identity with GAS PrsA1 and PrsA2 among the analyzed *Streptococcus* species (Supplementary Fig. 3b, c), providing additional evidence for the distinct cell cytotoxicity profiles observed between GAS PrsA and SsPrsA, thus affirming the safety of GAS PrsA as vaccine candidate. Of note, the highest level of similarity to GAS PrsA was observed with *S*. *dysgalactiae*, an emerging human pathogen capable of causing diseases similar to GAS, including cellulitis, necrotizing soft tissue infections, and streptococcal toxic shock syndrome^75–77^. This substantial similarity suggests that immunization with GAS PrsA1 and PrsA2 may offer potential advantages in reducing infections caused by *S. dysgalactiae*. However, further experiments are required to validate and explore this hypothesis.

In conclusion, our study highlights the potential of PrsA as a promising universal vaccine candidate against GAS infection. Further research is needed to optimize PrsA immunization efficacy and gain a better understanding of adjuvant mechanisms, which could expedite the development of a GAS vaccine in the future.

## Methods

### Bacterial strains

GAS clinical isolate A20 (*emm*1) and 4063-05 (*emm*4), as well as isogenic *prsA1/A2* deletion mutants generated in both backgrounds were previously described^18^. We enrolled 27 non-duplicated clinical isolates collected from the conventional laboratory of National Taiwan University Hospital during 2017–2020 for *emm* typing (Supplementary Table 1). The *emm* type of clinical isolates was determined according to CDC protocol (https://www.cdc.gov/streplab/groupa-strep/emm-background.html). All GAS strains were cultured in THY broth (Todd-Hewitt broth with 2% yeast extract) at 37 °C without shaking.

### GAS RNA isolation and qRT-PCR

To determine *prsA* expression levels in response to human serum treatment, log phase GAS was treated with RPMI 1640 medium (Mock) or RPMI 1640 medium with 10% normal human serum (NHS) for 30 min, resuspended in TRI reagent^®^ and mechanically lysed with MagNA Lyser (Roche). RNA was isolated using the Direct-Zol RNA MiniPrep kit (Zymo Research) with contaminating DNA being removed with additional DNase treatments (Qiagen) and transcribed to cDNA by PrimeScript^TM^ RT reagent Kit (Takara). Quantitative RT-PCR was performed on CFX96 Touch^TM^ Real-time PCR detection system (Bio-Rad) using the qPCRBIO SyGREEN blue mix (PCR Biosystems) according to the manufacturer’s instruction. Primers used for experiments were as follows: *prsA1*, TTATTGTGCAGCTGGCTCTT, TGAAAGTTCAACAACCAGCG; *prsA2*, AGGTTTTGCAAACTGGGCTAA, AAAGACATCATTGTGACTGGC; *recA*, GGATAACCACCAGCTCCAAG, AAGCTCTTGATGATGCTTTGAA; *gyrsA*, GAAGTGATCCCTGGACCTGA, CCCGACCTGTTTGAGTTGTT; *proS*, GGTGGTTCTTGACAAGTCTAT, CTGCCAAGGCATCTTCAGCA.

### Western blot analysis of PrsA1 and PrsA2 in GAS membrane protein extraction

For membrane protein preparation, overnight grown GAS was resuspended in KPN buffer (20 mM potassium phosphate, 140 mM NaCl [pH 7.5]) containing lysozyme (Sigma, 400 μg/ml), RNase (Sigma, 6 μg/ml), DNase (Sigma, 6 μg/ml) and protease inhibitor cocktail (Roche), incubated at room temperature for 10 min, and sonicated with *Vibra-Cell*™ VX130 (Sonic & Materials) to completely disrupt bacterial cells. The bacterial lysates were first centrifuged at 10,000 × *g* to remove cell debris, and the supernatants were collected and centrifuged at 44,000 rpm (type 100 Ti rotor, Beckman) to precipitate the crude membrane fraction. The crude membrane (1.5 μg) was resuspended in 1% NP-40 lysis buffer, separated on 10% SDS-PAGE, transferred to the PVDF membrane, probed with antibodies recognizing PrsA1 and PrsA2 (1 μg/ml), respectively, and visualized with a Li-Cor Odyssey scanner after addition of IRDye^®^ 680RD-conjugated secondary antibodies (1:10,000 dilution, Li-Cor, Cat. No. 926-68071).

### Preparation of recombinant proteins and hyperimmune sera

Recombinant PrsA1 and PrsA2 proteins and antibodies recognizing PrsA1 and PrsA2 were generated as previously described with slight modification^18^. Briefly, the open-reading frames (ORFs) of the *prsA1* and *prsA2* gene without the predicted signal-peptide coding sequence were amplified and cloned into pET15b and transformed into *E. coli* (ED3) cells. The target protein synthesis was induced with 1 mM isopropyl-thio-β-D-galactoside (IPTG) for 4 h at 37 °C. Bacterial cells were sonicated and centrifuged, and the resulted supernatant was subjected to purification with Ni-NTA resins (Qiagen) and size exclusion chromatography with HiLoad^®^ 16/600 Superdex 200 (Cytiva). The protein yield is around 5 mg from 500 ml bacterial culture after all the purification procedures and the purified proteins remain stable showing only one major peak in the size-exclusion chromatography, indicating no obvious degradation and aggregation (Supplementary Fig. 4a, b). The purity of proteins was examined by SDS-PAGE and Coomassie blue staining (Supplementary Fig. 4c, d), showing more than 95% for each protein. Hyperimmune sera to PrsA1 and PrsA2 were obtained from New Zealand white rabbits after 4 times of intradermal immunization at a 2-week interval with 200 μg of purified recombinant proteins mixed with complete or incomplete Freund’s adjuvant (LTK Biolaboratories Company, Taiwan). All hyperimmune sera had titers of more than 1:625,000 as measured by western blot against 0.1 μg of immunized protein. Pre-immune sera collected from the same rabbit before PrsA immunization, or rabbit antibodies purified from these pre-immune sera were served as negative controls for the following experiments. To purify the rabbit anti-PrsA1 and anti-PrsA2 antibodies, the hyperimmune sera were 10-fold diluted with PBS, incubated with 0.5 ml of rProtein A resins (Cytiva) for 16 h at 4 °C. The resin was washed with 10 ml of PBS 3 times and bound antibodies were eluted with 5 ml of 0.1 M glycin-HCl, pH3.0. The eluents were immediately neutralized with 0.5 ml of 1 M Tris-HCl, pH8.0 and exchanged to PBS by Amicon® Ultra (30 kDa cutoff, Merck). The specificity of anti-PrsA1 (Supplementary Fig. 4e) and anti-PrsA2 antibodies (Supplementary Fig. 4f) was confirmed by probing GAS crude membrane fractions extracted from wild type (WT), *prsA*-deficient mutant (Δ*prsA*) and *prsA*-complemented strains (CΔ*prsA*) generated in M1 A20 and M4 4063-05 background.

### Western blot analysis for heart lysate cross-reactivity

Recombinant M1 proteins and primary human adult heart lysates were kindly provided by Dr. Mark Walker (University of Queensland) and Dr. Kai-Chien Yang (National Taiwan University), respectively. Human right heart atrium was lysed in RIPA buffer (0.5% sodium deoxycholate, 0.1% sodium dodecyl sulfate, and 1% NP-40 in PBS) with protease inhibitors, passed several times through a 21-gauge needle, and centrifuged at 20,000 × *g* for 30 min at 4 °C. The collected protein supernatant was separated on 7.5% SDS-PAGE gel, transferred to the PVDF membrane. The membrane blot was blocked with Intercept^®^ blocking buffer (Li-Cor) and probed with rabbit antisera (1:10,000 dilution) generated against PrsA1 and PrsA2 proteins^18^ and mouse antisera (1:1,000 dilution) generated against GAS M1 proteins (kindly provided by Dr. Mark Walker). After extensive washes, IRDye^®^ 800CW- or IRDye^®^ 680RD-conjugated secondary antibodies (1:10,000 dilution, Li-Cor, Cat. No. 925-32212 and 926-68071) were added and the resulted near-infrared fluorescent signals were visualized and recorded with a Li-Cor Odyssey scanner. The uncropped and unprocessed images used to generate Fig. 1d, e are shown in Supplementary Fig. 6.

### Whole bacterial cell ELISA

Overnight grown GAS cultures (~ OD_600_ of 0.9–1) were washed with PBS and concentrated to OD_600_ of 6 in PBS. Nunc MaxiSorp plates (Thermo) were coated with 50 µl/well of bacterial cell suspension, blocked with 3% BSA/PBS for 1 h, and incubated with rabbit antibodies (3 μg/ml) specific to either PrsA1 or PrsA2, or rabbit antibodies purified from pre-immune rabbit sera for 2 h, followed by HRP-conjugated donkey anti-rabbit IgG (BioLegend, Cat. No. 406401; 1:5,000 dilution in 3% BSA/PBS with 0.05% Tween-20) for 1 h. Plates were washed, developed with TMB substrate (BioLegend) for 30 min in the dark, stopped with 0.1 N H_2_SO_4_, and measured at 450 nm. The OD_450_ readings of PrsA1- and PrsA2-specific signals were then subtracted from the OD_450_ readings of pre-immune IgG control, and normalized to the values obtained from M1 GAS A20 strain.

### Opsonophagocytic killing (OPK) assay

Neutrophils were isolated from blood drawn from healthy donors using PolymorphPrep (Axis-Shield), and adjusted to a final concentration of 2 × 10^6^ cells/ml. Mid-log phase GAS (2 × 10^5^) were incubated with 50 μl of PrsA antisera or naïve rabbit sera (pooled sera from two rabbits before PrsA immunization) for 30 min at 37 °C. Neutrophils were preincubated with 4% baby rabbit complement (Bio-Rad, Cat. No. C12CA) for 10 min and added to bacteria at a MOI of 1, briefly centrifuged to ensure contact, and incubated for 30 min at 37 °C. After incubation, samples were serially diluted in sterile water to lyse cells and plated onto THY agar. Sera from pre-immunized rabbits were used as control for the baseline bacterial killing of neutrophils. At minimum, each serum or serum combination was tested in triplicates with 2–3 independent experiments to ensure statistical confidence. Bacterial survival was calculated by dividing the number of surviving bacterial CFUs in PrsA antisera-treated group by the number of bacterial CFUs recovered from the naïve sera-treated group.

### Ex vivo infection in human whole blood

In total, 10^4^ CFU of mid-log phase GAS were incubated with heparinized whole human blood in the presence of 0.5 μg of control rabbit antibodies, PrsA1 antibodies, PrsA2 antibodies or a combination of both PrsA1 and PrsA2 antibodies at 37 °C for 90 min. Surviving bacteria were enumerated by plating in triplicate onto THY plates. Bacterial survival was calculated by dividing the number of surviving bacterial CFUs in anti-PrsA antibody-treated group by the number of bacterial CFUs recovered from the control antibody-treated group.

### Passive transfer of immune serum and GAS challenge

Cohorts of 6-week-old female ICR mice (*n* = 14–25) were intraperitoneally administered with 200 μl of one of the following: pre-immune sera, PrsA1 antisera, PrsA2 antisera or a combination of both PrsA1 and PrsA2 antisera. Two hours after the antisera administration, the mice were intraperitoneally challenged with 4–8 × 10^7^ CFU of M1 GAS (strain NTU24) and closely monitored for survival for 6 days.

### Active immunization with CFA/IFA-adjuvanted PrsA and GAS challenge

Cohorts of 5-week-old ICR female mice were intramuscularly immunized on day 0 with 20 μg of PrsA1 and 20 μg of PrsA2 (*n* = 23) or 20 μg M1 (*n* = 4) emulsified 1:1 in complete Freund’s adjuvant (CFA, Sigma). To control for the effect of the adjuvant, parallel cohorts of mice were given control OVA proteins in CFA (*n* = 23) or PBS control (*n* = 8). PrsA, M1 and OVA groups received one boost with incomplete Freund’s adjuvant (IFA) emulsified proteins on day 21. Serum samples were collected 2 weeks after the final boost to examine PrsA-specific IgG titers by ELISA. Three weeks after the final boost, immunized mice were challenged with 5–6 × 10^7^ CFUs of M1 GAS (strain NTU24) and closely monitored for survival for 5 days. For bacterial burden measurements, immunized mice were challenged with sublethal doses of 2–3 × 10^7^ CFUs of M1 GAS (strain NTU24). All mice were euthanized 18 h post-infection by CO_2_ asphyxiation to harvest the spleen, kidney and blood. Spleens and kidneys were homogenized in 1 ml of PBS by MagNA Lyser (Roche) with 1 mm zirconium beads (BioSpec). Bacterial counts in blood and tissue homogenates were determined by plating serial dilutions.

### Active immunization with alum-adjuvanted PrsA and GAS challenge

Cohorts of 5-week-old female ICR mice (*n* = 10) were subcutaneously immunized three times at 2-week interval (days 0, 14, 28) with 20 μg of PrsA1 and 20 μg of PrsA2 absorbed on Alhydrogel® (Invivogen). To control for the effect of the adjuvant, parallel cohorts of mice were given control OVA proteins in alum. Serum samples were collected 2 weeks after the final boost, and PrsA-specific IgG titers were measured by ELISA. Two weeks after the final boost, immunized mice were challenged with 5–6 × 10^7^ CFUs of M1 GAS (strain NTU24) and closely monitored for survival for 6 days.

### PrsA-specific IgG titer measurement

Sera antibody titers to PrsA recombinant proteins were quantified using ELISA. Briefly, recombinant PrsA1 and PrsA2 proteins (1 μg/ml in 50 mM sodium carbonate/bicarbonate buffer, pH9.5) were coated onto Nunc MaxiSorp plates (Thermo) overnight at 4 °C. Wells were washed with PBST (phosphate-buffered saline with 0.05% Tween 20, pH 7.4), blocking with 5% skim milk, followed by addition of 3-fold serially diluted serum samples in 0.5% skim milk/PBST. After washing, HRP-conjugated secondary antibody (Cell Signaling Technology, Cat. No. 7076S) was applied at 1:5,000 dilution. Plates were developed with TMB substrate (BioLegend) for 20 min, stopped with 0.1 N H_2_SO_4_, and measured at 450 nm. IgG titers were defined as the highest dilution of serum for which OD_450_ exceeded the mean OD_450_ of negative control plus 3 standard deviations. To analyze the titers of PrsA-specific IgG subclass, HRP-conjugated goat anti-mouse Ig isotype antibodies (SouthernBiotech, IgG1, Cat. No. 1073-05; IgG2b, Cat. No. 1093-05; IgG2c, Cat. No. 1077-05 and IgG3, Cat. No. 1103-05; 1:8,000 dilution for IgG1 and 1:5,000 dilution for IgG2b, IgG2c and IgG3) were used to detect the levels of PrsA-bound IgG subclass in 96-well plates as described earlier. The ratios of IgG1/IgG2b, IgG1/IgG2c and IgG1/IgG3 were determined by dividing the titer of PrsA-specific IgG1 by the titer of PrsA-specific IgG2b, IgG2c and IgG3, respectively.

### Quantification of C3b deposition

10^7^ CFU of mid-log phase GAS were washed, resuspended in HEPES++/0.1% BSA (20 mM HEPES [pH7.4], 5 mM CaCl_2_, 2.5 mM MgCl_2_, 140 mM NaCl, 0.1% BSA), and incubated in 1% mouse sera for 20 min at 37 °C. After washing, samples were incubated with FITC-conjugated goat anti-complement C3 antibody (MP Biomedicals Cat. No. 55167) and analyzed by FACS Calibur® with Cell Quest software.

### Analysis of T cell responses by intracellular cytokine staining (ICS) and ELISA

Mouse spleens were harvested 7 days after the final immunization, smashed between the frosted ends of two glass slides, filtered through a cotton-plugged Pasteur pipette to remove cell debris. The erythrocytes were lysed by ACK lysis buffer (10 mM KHCO_3_, 0.1 mM EDTA, 155 mM NH_4_Cl). The resulted splenocytes were washed, resuspended in cRPMI (10% FBS, 0.1 mM non-essential amino acid, 1 mM penicillin-streptomycin, 1 mM sodium pyruvate, 2 mM L-glutamine, 10 mM HEPES and 0.05 mM 2-mercaptoethanol), seeded into 96-well plates (10^6^ cells/well), and stimulated with PMA (20 ng/ml) plus ionomycin (1 μg/ml), OVA proteins (5 μg/ml for ICS and 1 μg/ml for ELISA), or PrsA proteins (5 μg/ml for ICS and 1 μg/ml for ELISA) and incubated in cRPMI supplemented with or without brefeldin A (5 μg/ml) at 37 °C for ICS (6 h) and ELISA analysis (72 h), respectively. For ICS, stimulated cells were labeled with a cocktail containing anti-mouse CD3-APC/Cy7 antibody (BioLegend, Cat. No. 100329), CD4-BV421 antibody (BioLegend, Cat. No. 100543), and CD8-PE Dazzle 594 antibody (BioLegend, Cat. No. 100761). Following fixation with 4% paraformaldehyde and permeabilization with permeabilization buffer (Invitrogen), cells were labeled with a cocktail containing anti-mouse IL-4-APC (BioLegend, Cat. No. 504105), anti-mouse IFN-γ-PE antibody (BioLegend, Cat. No. 505807) and anti-mouse TNF-α-FITC antibody (BioLegend, Cat. No. 506303). After washing, cells were acquired by a Cytek^®^ Northern Lights^TM^ flow cytometer (Cytek Biosciences) and analyzed by FlowJo v10 (Tree Star Inc.). The levels of secreted cytokines in culture supernatants were evaluated using standard sandwich ELISA for IL-4 and IFN-γ (BioLegend) according to the manufacturer’s instructions.

### Ethical statements

Human blood was drawn from healthy donors with written informed consents and de-identification, as approved by the NTU University Human Research Ethics Committee (IRB 202012045RIND). The studies on patient samples with written informed consents were approved by the Institutional Review Board committee of National Cheng Kung University Hospital (No. ER-98-287, A-ER-103-394, and A-ER-106-473). All the animal experiments were performed in accordance with relevant guidelines and regulations approved by the National Taiwan University College of Medicine Animal Care and Use Committee (IACUC 20201110 and 20210293).

### Statistical analysis

Statistical tests were performed using GraphPad Prism version 9 software (GraphPad Software, Inc.). The statistical analyses used to determine the significance of group differences were indicated in the legends. Data presented here were combined, normalized and expressed as means ± SD except somewhere indicated, and *p* values < 0.05 were considered statistically significant for all tests.

### Reporting summary

Further information on research design is available in the Nature Research Reporting Summary linked to this article.

### Supplementary information

Supplementary Material

Reporting Summary

## Data Availability

All relevant data are presented in this paper. If any more information is needed, data are available from the corresponding author upon reasonable request.
