# Supplementary material for: Conserved molecular chaperone PrsA stimulates protective immunity against group A Streptococcus
Source: NPJ Vaccines. 2024 Feb 26;9:46. doi: 10.1038/s41541-024-00839-7 (PMC10897429; doi:10.1038/s41541-024-00839-7)
Supplement: Supplementary file 1 — Supplementary Material [file 41541_2024_839_MOESM1_ESM.pdf]

Supplementary Figure 1

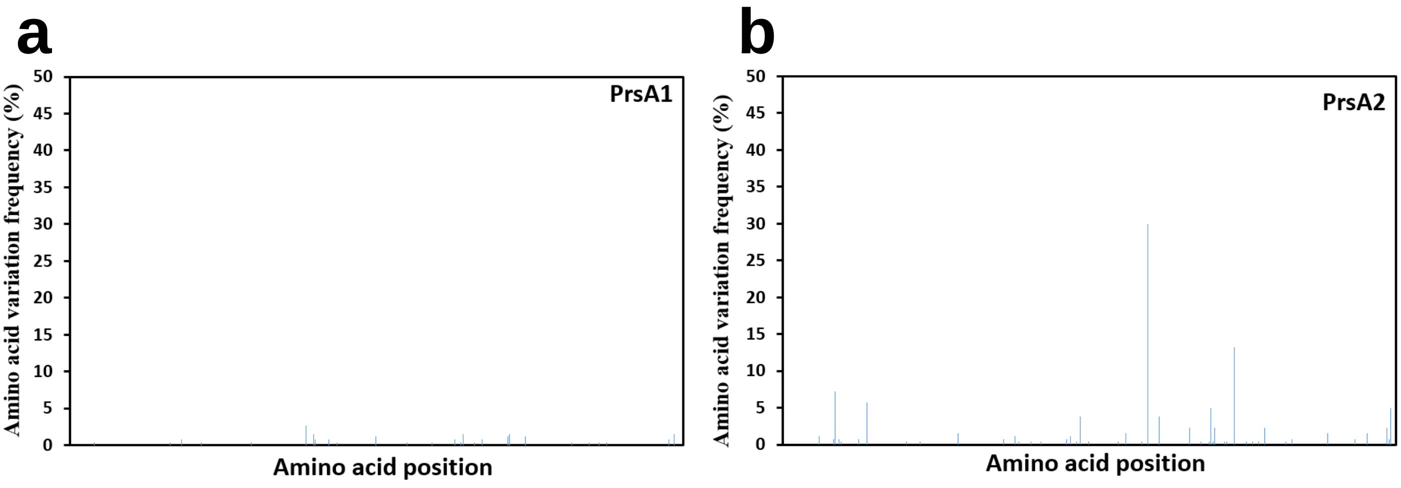

Supplementary Figure 1. Amino acid variations in PrsA1 and PrsA2 across different *emm*-type GAS strains. The nucleotide sequences of PrsA1 and PrsA2 were extracted from 264 publicly available GAS genomes, translated into amino acid sequences, aligned with NCBI COBALT and visualized using Multiple Sequence Alignment Viewer (MSA). The Frequency of amino acid variations of PrsA1 and PrsA2 was shown in (a) and (b), respectively. The strain name and genome accession number were included in the Supplementary table 2.

Supplementary Figure 2

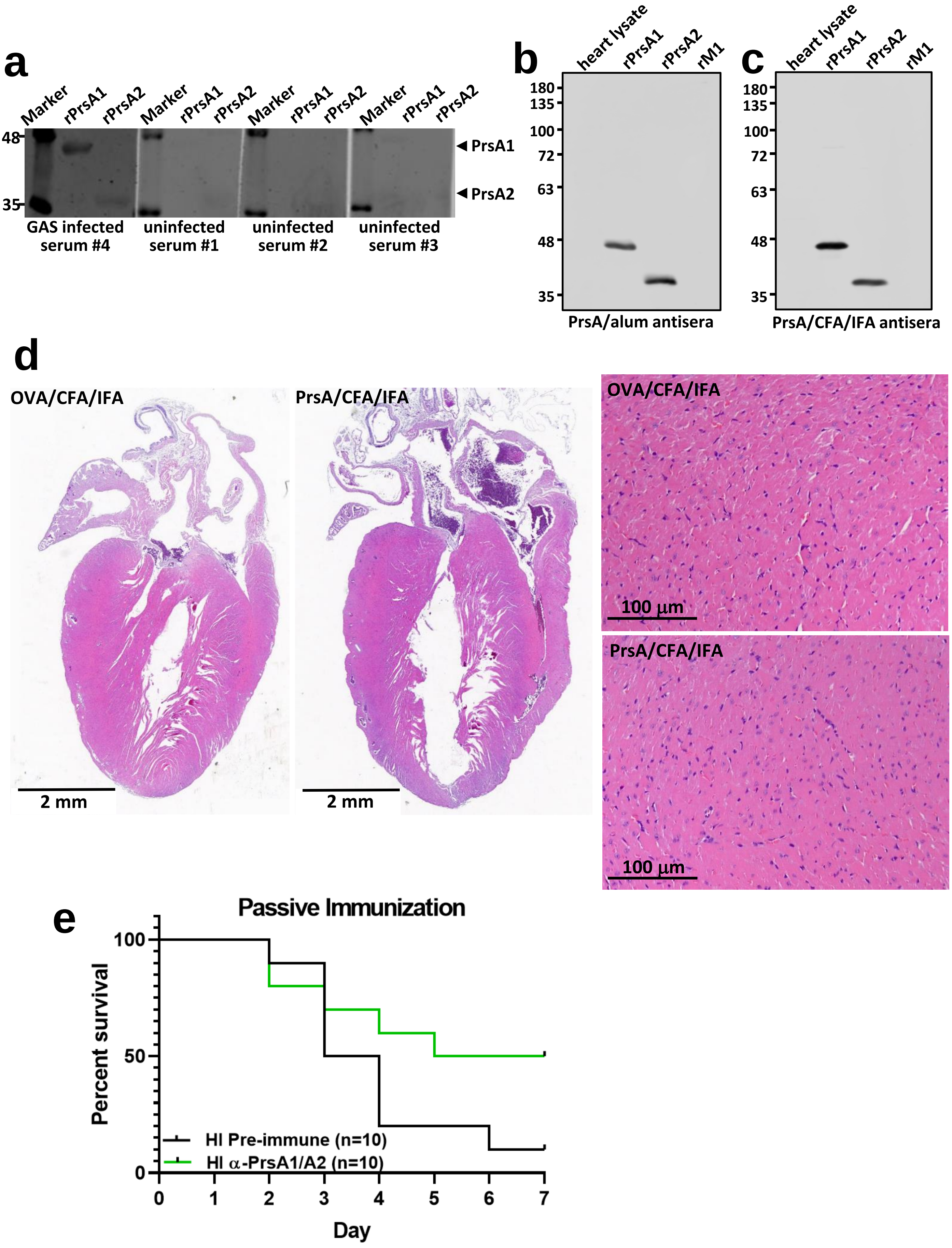

Supplementary Figure 2. Immunogenicity and toxicity of PrsA1 and PrsA2, and the passive protective immunity of PrsA1 and PrsA2 antisera against GAS infection. (a) Recombinant PrsA1 and PrsA2 proteins (1 μg) were separated by SDS-PAGE, and probed with antisera collected from GAS-infected mice (1:500 dilution) or pre-immune sera (1:500 dilution). The GAS-infected serum #4 (the same serum shown in Fig. 1d) served as positive control. Human heart lysate (20 μg), recombinant PrsA1, PrsA2, and M1 proteins (each 0.05 μg) were separated by SDS-PAGE, and probed with mouse antisera (1:1,000 dilution) collected 1 week post last-immunization from PrsA/alum (b) and PrsA/CFA/IFA (c) immunized mice. (d) Histological changes in cardiac tissues following PrsA immunization. ICR mice were immunized on days 0 and 21 with OVA/CFA/IFA or PrsA/CFA/IFA. Hearts were harvest 1 week post last-immunization, fixed in formalin, embedded in paraffin, sectioned and stained with haematoxylin and eosin (H&E). Images magnified at x50 (left) and x200 (right). (e) Kaplan-Meier survival curve of mice. Groups of female ICR mice (n = 10) were intraperitoneally injected with 56 °C heat-inactivated rabbit PrsA1/A2-specific antisera or with 56 °C heat-inactivated rabbit pre-immune sera. Two hours later, the mice were challenged intraperitoneally with lethal dose of M1 GAS strain NTU24 and monitored for survival for a week.

Supplementary Figure 3

a

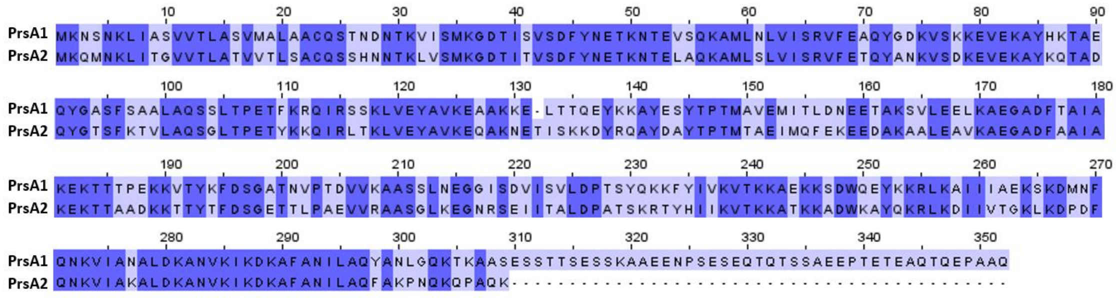

b

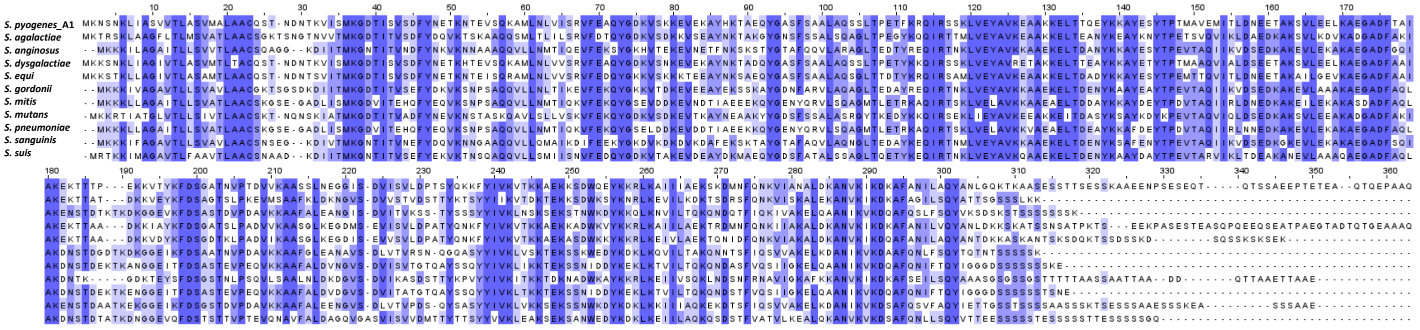

Percent animo acid identity matrix of GAS PrsA1 with other Streptococcus species											
	S.pyogenes_A1	S.agalactiae	S.anginosus	S.dysgalactiae	S.equi	S.gordonii	S.mitis	S.mutans	S.pneumoniae	S.sanguinis	S.suis
S.pyogenes_A1	100.00	57.47	47.40	78.16	71.73	46.56	44.16	52.41	44.01	44.41	45.79
S.agalactiae	57.47	100.00	51.64	59.74	61.04	53.27	48.69	60.66	47.39	51.97	54.58
S.anginosus	47.40	51.64	100.00	49.03	50.32	72.96	65.81	50.00	65.27	73.40	62.50
S.dysgalactiae	78.16	59.74	49.03	100.00	76.36	47.54	44.48	55.45	44.01	44.21	46.54
S.equi	71.73	61.04	50.32	76.36	100.00	48.84	42.86	52.29	42.07	47.19	48.44
S.gordonii	46.56	53.27	72.96	47.54	49.84	100.00	64.61	49.83	63.96	74.59	63.84
S.mitis	44.16	48.69	65.81	44.48	42.86	64.61	100.00	48.04	93.59	60.00	55.95
S.mutans	52.41	60.66	50.00	55.45	52.29	49.83	48.04	100.00	49.19	47.83	49.69
S.pneumoniae	44.01	47.39	65.27	44.01	42.07	63.96	93.59	49.19	100.00	60.13	56.73
S.sanguinis	44.41	51.97	73.40	44.21	47.19	74.59	60.00	47.84	60.13	100.00	58.51
S.suis	45.79	54.48	62.50	46.54	48.44	63.84	55.95	49.69	56.73	58.51	100.00

Supplementary Figure 3

c

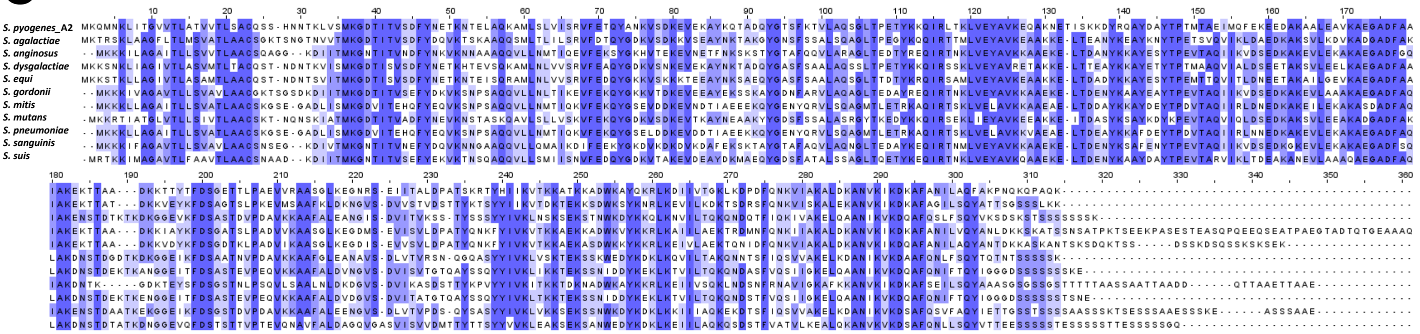

Percent animo acid identity matrix of GAS PrsA2 with other Streptococcus species											
	S.pyogenes_A2	S.agalactiae	S.anginosus	S.dysgalactiae	S.equi	S.gordonii	S.mitis	S.mutans	S.pneumoniae	S.sanguinis	S.suis
S.pyogenes_A2	100.00	54.55	43.23	68.51	63.31	43.28	39.67	53.44	40.66	42.57	44.26
S.agalactiae	54.55	100.00	51.64	59.74	61.04	53.27	48.69	60.66	47.39	51.97	54.58
S.anginosus	43.23	51.64	100.00	49.03	50.32	72.96	65.81	50.00	65.27	73.40	62.50
S.dysgalactiae	68.51	59.74	49.03	100.00	75.98	47.54	44.48	55.26	44.01	44.71	46.11
S.equi	63.31	61.04	50.32	75.98	100.00	49.84	42.86	53.09	42.07	46.89	48.42
S.gordonii	43.28	53.27	72.96	47.54	49.84	100.00	64.61	49.83	63.96	74.59	63.84
S.mitis	39.67	48.69	65.81	44.48	42.86	64.61	100.00	48.04	93.59	60.00	55.95
S.mutans	53.44	60.66	50.00	55.26	53.09	49.83	48.04	100.00	49.19	48.00	49.53
S.pneumoniae	40.66	47.39	65.27	44.01	42.07	63.96	93.59	49.19	100.00	60.13	56.73
S.sanguinis	42.57	51.97	73.40	44.71	46.89	74.59	60.00	48.00	60.13	100.00	58.51
S.suis	44.26	54.58	62.50	46.11	48.42	63.84	55.95	49.53	56.73	58.51	100.00

d

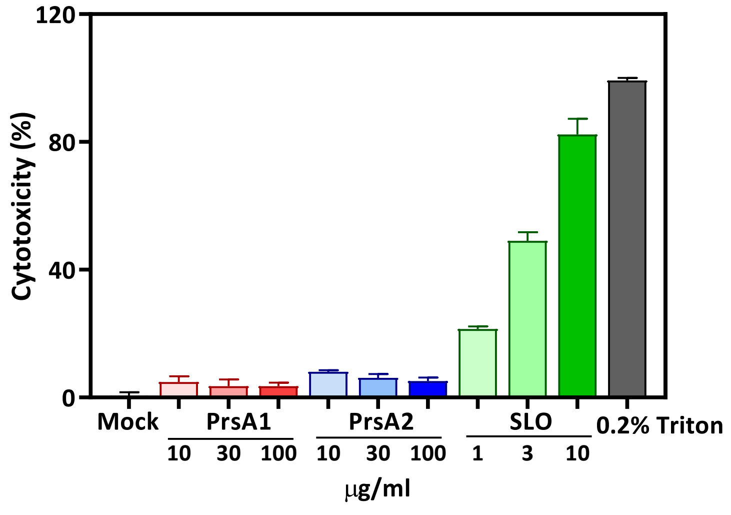

Supplementary Figure 3. Amino acid comparison and cell cytotoxicity of GAS PrsA. (a) Alignment of the amino acid sequences of GAS PrsA1 and PrsA2. Dark purple shading highlights identical amino acids, while light purple indicates non-identical ones. The amino acid sequence of PrsA1 (b) and PrsA2 (c) were aligned with PrsA from different *Streptococcus* species by Clustal Omega and visualized using Jalview. Darker purple indicates a higher percentage identity between *Streptococcus* species. The percent identity matrix was shown in the lower panel. (d) Evaluation of the cytotoxicity of GAS PrsA1 and PrsA2. RAW264.7 macrophages (10^5 cells) were treated with purified PrsA1, PrsA2 and streptolysin O (SLO) at different concentrations for 2 h, using PBS and 0.2% Triton X-100 as negative and positive controls, respectively. Cell viability was quantified by LDH cytotoxicity assay kit.

Supplementary Figure 4

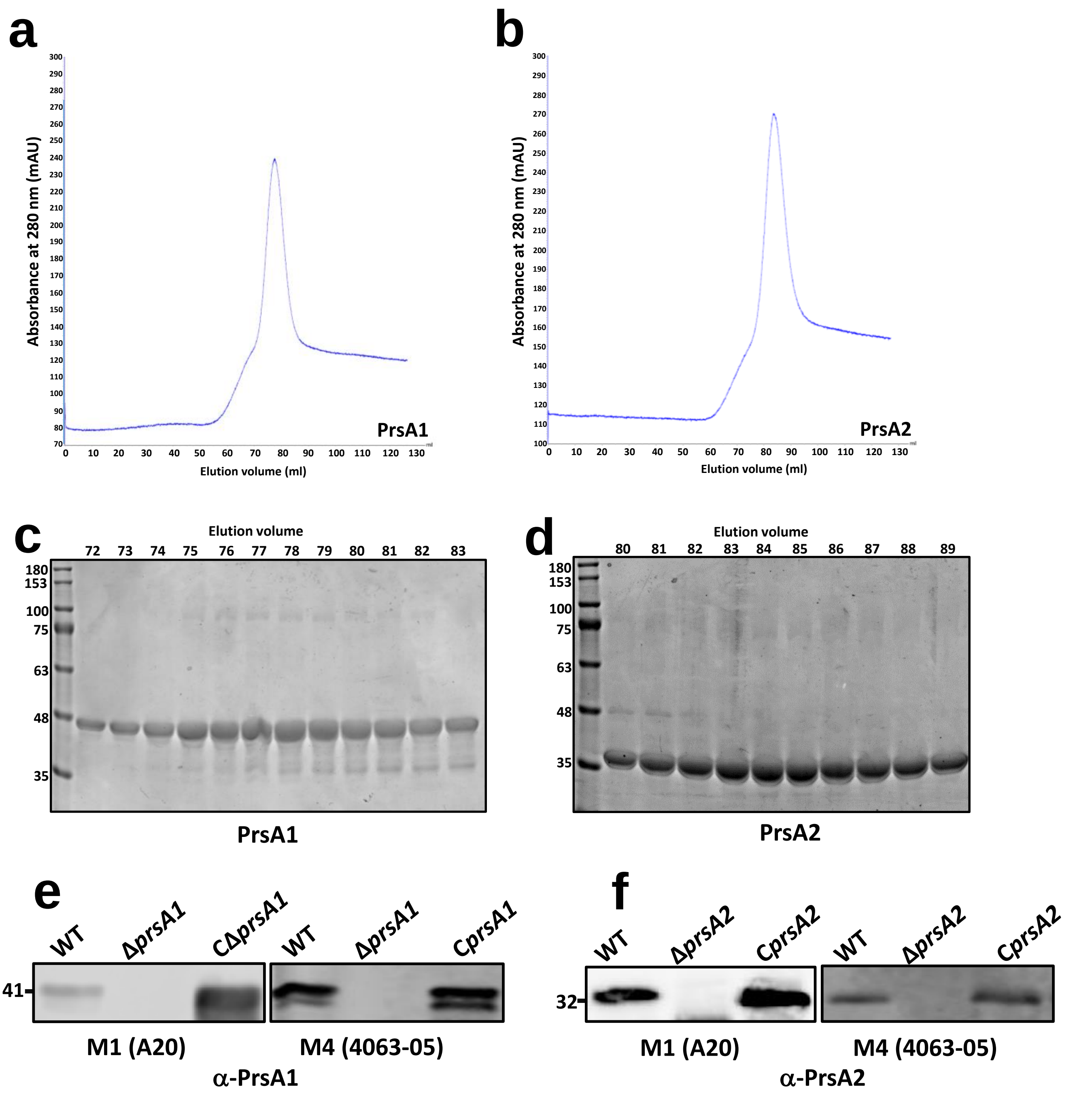

Supplementary Figure 4. Verification of the materials used in this study. (a-d) Purity and size homogeneity of purified GAS PrsA1 and PrsA2. Recombinant PrsA1 and PrsA2 were expressed in *E. coli* and purified by Ni-NTA affinity chromatography and Superdex 200 size-exclusion chromatography (SEC). SEC chromatograms for PrsA1 (a) and PrsA2 (b). The peak fractions were collected and separated on 10% SDS-PAGE followed by Coomassie blue staining for PrsA1 (c) and PrsA2 (d). (e) Crude membrane fractions collected from wild type (WT), *prsA1*-deficient mutant (Δ*prsA1*) and *prsA1*-complemented strains (CΔ*prsA1*) generated in M1 A20 and M4 4063-05 background were analyzed by Western blot with anti-PrsA1 antibodies (1:5,000 dilution). (f) Crude membrane fractions collected from wild type (WT), *prsA2*-deficient mutant (Δ*prsA2*) and *prsA2*-complemented strains (CΔ*prsA2*) generated in M1 A20 and M4 4063-05 background were analyzed by Western blot with anti-PrsA2 antibodies (1:5,000 dilution).

Supplementary Figure 5

a

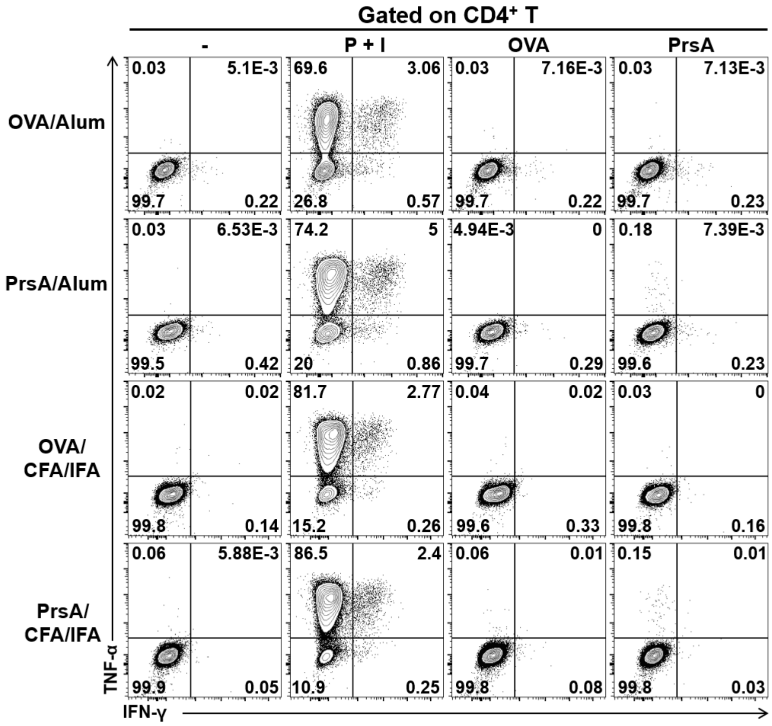

b

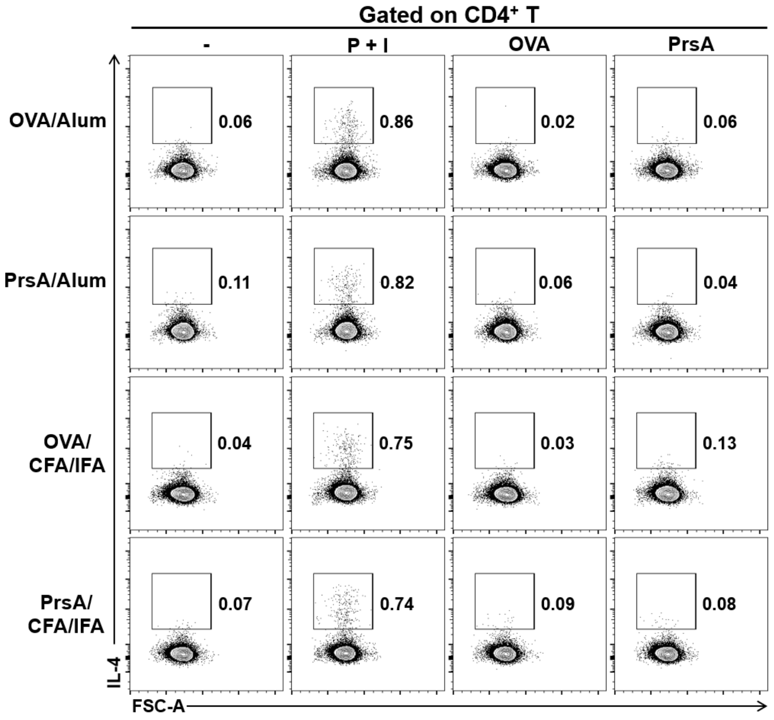

Supplementary Figure 5. The frequency of IFN-γ^{+}CD4^{+}, TNF-α^{+}CD4^{+}, and IL4^{+}CD4^{+} T cells. ICR mice were immunized on days 0 and 21 with OVA/alum, PrsA/alum, OVA/CFA/IFA or PrsA/CFA/IFA. Spleens were harvest one week post last-immunization. Total splenocytes (10^{6}) were stimulated with media alone (-), PMA (20 ng/ml) plus ionomycin (1 µg/ml) (P+I), OVA or PrsA proteins (5 µg/ml) for 6 h, and cytokine expression in CD4 T cells was evaluated by intracellular cytokine stain. (a) Representative plots of IFN-γ- and TNF-α-producing CD4^{+} T cells. (b) Representative plots of IL-4-producing CD4^{+} T cells.

Supplementary Figure 6

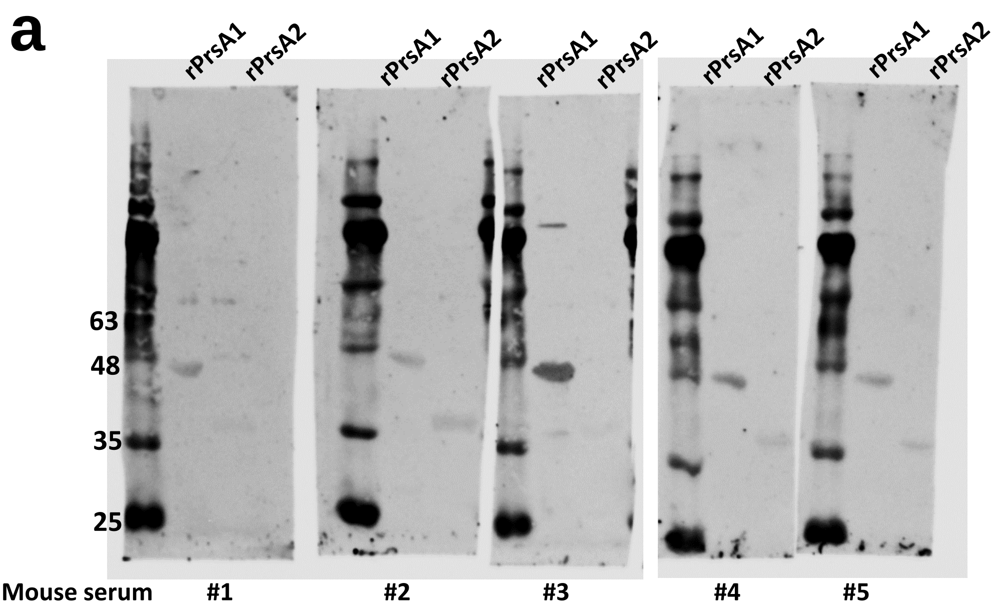

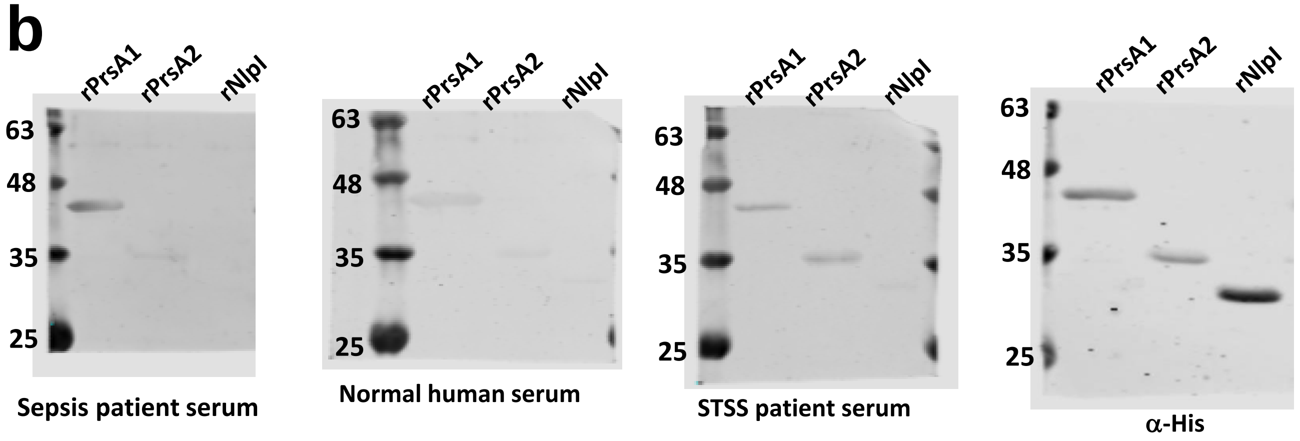

Supplementary Figure 6. Images used to generate western showing 1d and 1e. Uncropped and unprocessed images corresponding to Fig. 1d and Fig. 1e were shown in (a) and (b), respectively.

Supplementary Table 1. Clinical GAS strains used in this study

**Strain number**	**Site of isolation**	***emm* type**
NTU1	Skin pus	113
NTU2	Skin pus	77
NTU3	Blood	58.7
NTU5	Skin pus	49
NTU6	Skin pus	89
NTU7	Blood	58.7
NTU8	Skin pus	1
NTU9	Skin pus	58
NTU10	Blood	12.68
NTU11	Blood	75
NTU12	Blood	98.1
NTU15	Blood	77
NTU16	Throat swab	1
NTU17	Throat swab	12
NTU22	Throat swab	12
NTU24	Blood	1
NTU25	Throat swab	12
NTU30	Skin pus	89
NTU32	Skin pus	124
NTU34	Throat swab	89
NTU36	Skin pus	58.7
NTU41	Throat swab	1
NTU43	Blood	49
NTU45	Sputum	6.82
NTU46	Skin pus	58
NTU47	Throat swab	12.19
NTU48	Throat swab	4

Supplementary Table 2. GAS strains used for PrsA alignment

**GAS accession number**
Sequence 1: CP049187.1_strain=TSPY1687_1
Sequence 2: CP009612.1_strain=HKU360_1
Sequence 3: CP055246.1_strain=TSPY767_1
Sequence 4: CP049697.1_strain=ABC3_1
Sequence 5: CP049687.1_strain=TSPY515_1
Sequence 6: CP049692.1_strain=ABC157_1
Sequence 7: CP049693.1_strain=ABC155_1
Sequence 8: CP049688.1_strain=ABC245_1
Sequence 9: CP049694.1_strain=ABC122_1
Sequence 10: CP049689.1_strain=ABC221_1
Sequence 11: CP049690.1_strain=ABC208_1
Sequence 12: CP049695.1_strain=ABC76_1
Sequence 13: CP049685.1_strain=TSPY806_1
Sequence 14: CP049691.1_strain=ABC199_1
Sequence 15: CP049696.1_strain=ABC25_1
Sequence 16: CP049686.1_strain=TSPY637_1
Sequence 17: CP007560.1_strain=NGAS743_1
Sequence 18: CP060643.1_strain=TSPY416_1
Sequence 19: CP060649.1_strain=TSPY270_1
Sequence 20: CP060645.1_strain=TSPY1349_1
Sequence 21: CP060642.1_strain=TSPY1312_1
Sequence 22: CP060646.1_strain=TSPY1026_1
Sequence 23: CP060647.1_strain=TSPY136_1
Sequence 24: CP072112.1_strain=M08500_1
Sequence 25: CP067010.1_strain=iGAS376_1
Sequence 26: CP067008.1_strain=iGAS426_1
Sequence 27: CP035429.1_strain=emmNA_1
Sequence 28: CP035436.1_strain=emm89.14_1
Sequence 29: LR130237.1_strain=SP444_1
Sequence 30: CP067009.1_strain=iGAS391_1
Sequence 31: CP049799.1_strain=1040_1
Sequence 32: CP051138.1_strain=4063-05_1
Sequence 33: LR590466.1_strain=NCTC8193_1
Sequence 34: CP041408.1_strain=37-97S_1
Sequence 35: AP023389.1_strain=KUN-0012590_1

Sequence 36: CP031738.1_strain=SP1336_1
Sequence 37: CP044093.1_strain=FDAARGOS_668_1
Sequence 38: CP047120.1_strain=1085_1
Sequence 39: CP035439.1_strain=emm77_1
Sequence 40: CP035443.1_strain=emm58_1
Sequence 41: CP035434.1_strain=emm75.1_1
Sequence 42: CP035452.1_strain=emm123_1
Sequence 43: CP035438.1_strain=emm22.8_1
Sequence 44: CP035447.1_strain=emm97.1_1
Sequence 45: CP035437.1_strain=emm78.3_1
Sequence 46: CP031770.1_strain=SASM4-Duke_1
Sequence 47: CP061132.1_strain=BSAC_bs1388_1
Sequence 48: CP061134.1_strain=BSAC_bs192_1
Sequence 49: CP061133.1_strain=BSAC_bs472_1
Sequence 50: CP061131.1_strain=BSAC_bs1802_1
Sequence 51: CP033908.1_strain=RLGH_1
Sequence 52: CP033907.1_strain=Duke-Large_1
Sequence 53: CP033767.1_strain=FDAARGOS_534_1
Sequence 54: LS483333.1_strain=NCTC12048_1
Sequence 55: LS483329.1_strain=NCTC12058_1
Sequence 56: LS483414.1_strain=NCTC13736_1
Sequence 57: LS483315.1_strain=NCTC12059_1
Sequence 58: LS483425.1_strain=NCTC13737_1
Sequence 59: LS483330.1_strain=NCTC8328_1
Sequence 60: LS483355.1_strain=NCTC12067_1
Sequence 61: LS483360.1_strain=NCTC10876_1
Sequence 62: LS483323.1_strain=NCTC8300_1
Sequence 63: LS483336.1_strain=NCTC12046_1
Sequence 64: LS483420.1_strain=NCTC13739_1
Sequence 65: LS483357.1_strain=NCTC8326_1
Sequence 66: LS483401.1_strain=NCTC10085_1
Sequence 67: CP028148.1_strain=TJ11-001_1
Sequence 68: CP119598.1_strain=1042_1
Sequence 69: CP119599.1_strain=1133_1
Sequence 70: CP119597.1_strain=1039_1
Sequence 71: CP119678.1_strain=1004_1
Sequence 72: CP013838.1_strain=MGAS11027_1

Sequence 73: CP013839.1_strain=MGAS23530_1
Sequence 74: CP116457.1_strain=1095_1
Sequence 75: HG316453.1_strain=H293_1
Sequence 76: NC_009332.1_strain=Manfredo_1
Sequence 77: NC_008024.1_strain=MGAS10750_1
Sequence 78: NC_008021.1_strain=MGAS9429_1
Sequence 79: CP097251.1_strain=21SPY7071_1
Sequence 80: CP014138.1_strain=MEW427_1
Sequence 81: LR134272.1_strain=NCTC12060_1
Sequence 82: NC_008023.1_strain=MGAS2096_1
Sequence 83: CP049800.1_strain=1043_1
Sequence 84: CP007537.1_strain=AP1_1
Sequence 85: CP008926.1_strain=ATCC19615_1
Sequence 86: CP007041.1_strain=STAB902_1
Sequence 87: CP008776.1_strain=5448_1
Sequence 88: CP007561.1_strain=NGAS596_1
Sequence 89: CP060638.1_strain=TSPY141_1
Sequence 90: CP060648.1_strain=TSPY383_1
Sequence 91: CP060644.1_strain=TSPY1309_1
Sequence 92: CP060641.1_strain=TSPY210_1
Sequence 93: CP077685.1_strain=M49-591_1
Sequence 94: CP070994.1_strain=M11318_1
Sequence 95: CP040997.1_strain=FDAARGOS_774_1
Sequence 96: CP035446.1_strain=emm68.2_1
Sequence 97: CP035449.1_strain=emm56_1
Sequence 98: CP035427.1_strain=emm74_1
Sequence 99: CP035440.1_strain=emm124_1
Sequence 100: CP035431.1_strain=emm105_1
Sequence 101: CP035435.1_strain=emm64.3_1
Sequence 102: CP035454.1_strain=emm54_1
Sequence 103: CP035445.1_strain=emm92_1
Sequence 104: CP035453.1_strain=emm100_1
Sequence 105: CP031630.1_strain=MGAS28271_1
Sequence 106: CP035441.1_strain=emm1_1
Sequence 107: LR130238.1_strain=HKU419_1
Sequence 108: CP065927.1_strain=emm9ST603_1
Sequence 109: AP023390.1_strain=KUN-0014944_1

Sequence 110: AP023387.1_strain=NIH34_1
Sequence 111: AP023388.1_strain=NIH35_1
Sequence 112: CP031636.1_strain=MGAS10826_1
Sequence 113: CP031634.1_strain=MGAS11108_1
Sequence 114: CP031628.1_strain=MGAS28330_1
Sequence 115: CP031627.1_strain=MGAS28360_1
Sequence 116: CP031622.1_strain=MGAS28686_1
Sequence 117: CP031640.1_strain=MGAS7888_1
Sequence 118: CP031639.1_strain=MGAS7914_1
Sequence 119: CP031635.1_strain=MGAS11052_1
Sequence 120: CP031632.1_strain=MGAS28078_1
Sequence 121: CP031633.1_strain=MGAS11115_1
Sequence 122: CP031631.1_strain=MGAS28191_1
Sequence 123: CP031625.1_strain=MGAS28533_1
Sequence 124: CP031619.1_strain=MGAS29284_1
Sequence 125: CP031637.1_strain=MGAS10786_1
Sequence 126: CP031626.1_strain=MGAS28386_1
Sequence 127: CP031624.1_strain=MGAS28650_1
Sequence 128: CP031618.1_strain=MGAS29326_1
Sequence 129: CP031638.1_strain=MGAS8347_1
Sequence 130: CP031620.1_strain=MGAS29064_1
Sequence 131: CP031629.1_strain=MGAS28278_1
Sequence 132: CP031623.1_strain=MGAS28669_1
Sequence 133: CP031617.1_strain=MGAS29409_1
Sequence 134: LR130240.1_strain=PS006_1
Sequence 135: CP043530.1_strain=MGAS2221_1
Sequence 136: NC_021807.1_strain=HSC5_1
Sequence 137: NC_020540.2_strain=M1-476_1
Sequence 138: CP045930.1_strain=AUSMDU00010539_1
Sequence 139: CP028841.1_strain=CCUG4207_1
Sequence 140: CP036531.1_strain=STAB10048_1
Sequence 141: CP036530.1_strain=STAB09023_1
Sequence 142: CP035448.1_strain=emm70_1
Sequence 143: LR031521.1_strain=S119_1
Sequence 144: CP033815.1_strain=FDAARGOS_514_1
Sequence 145: AP019548.1_strain=10-85_1
Sequence 146: CP032700.1_strain=TSPY556_1

Sequence 147: CP033336.1_strain=TSPY165_1
Sequence 148: NC_007296.2_strain=MGAS6180_1
Sequence 149: CP035455.1_strain=emm197_1
Sequence 150: CP035444.1_strain=emm90.5_1
Sequence 151: CP032665.1_strain=MGAS27961_1
Sequence 152: CP032666.1_strain=MGAS28085_1
Sequence 153: NC_018936.1_strain=A20_1
Sequence 154: CP064364.1_strain=PartK-Spyogenes-RM8376_1
Sequence 155: AP018337.1_strain=KS030_1
Sequence 156: CP027771.1_strain=DMG1800716_1
Sequence 157: LS483359.1_strain=NCTC12068_1
Sequence 158: LS483442.1_strain=NCTC8370_1
Sequence 159: LS483307.1_strain=NCTC5163_1
Sequence 160: LS483332.1_strain=NCTC12696_1
Sequence 161: LS483335.1_strain=NCTC8332_1
Sequence 162: LS483327.1_strain=NCTC12069_1
Sequence 163: LS483340.1_strain=NCTC12062_1
Sequence 164: LS483394.1_strain=NCTC10880_1
Sequence 165: LS483379.1_strain=NCTC10874_1
Sequence 166: LS483322.1_strain=NCTC12066_1
Sequence 167: LS483345.1_strain=NCTC8231_1
Sequence 168: LS483321.1_strain=NCTC8314_1
Sequence 169: LS483389.1_strain=NCTC10879_1
Sequence 170: LS483430.1_strain=NCTC12044_1
Sequence 171: LS483432.1_strain=NCTC13745_1
Sequence 172: LS483320.1_strain=NCTC5164_1
Sequence 173: LS483334.1_strain=NCTC12050_1
Sequence 174: LS483337.1_strain=NCTC12047_1
Sequence 175: LS483407.1_strain=NCTC13744_1
Sequence 176: CP014027.2_strain=FDAARGOS_149_1
Sequence 177: CP028140.1_strain=NGAS979_1
Sequence 178: CP023769.1_strain=HarveyGAS_1
Sequence 179: CP021640.1_strain=JS12_1
Sequence 180: AP017629.1_strain=JMUB1235_1
Sequence 181: AP014596.1_strain=M3-b_1
Sequence 182: CP022354.1_strain=GUR_1
Sequence 183: CP022206.1_strain=GURSA1_1

Sequence 184: NC_017596.1_strain=Alab49_1
Sequence 185: LN831034.1_strain=NCTC8198_1
Sequence 186: CP011535.2_strain=M28PF1_1
Sequence 187: CP011069.1_strain=STAB09014_1
Sequence 188: CP121250.1_strain=1044_1
Sequence 189: CP013840.1_strain=MGAS27061_1
Sequence 190: CP060267.1_strain=SP1448_1
Sequence 191: CP060266.1_strain=SP1450_1
Sequence 192: CP060270.1_strain=SP1384_1
Sequence 193: CP060268.1_strain=SP1426_1
Sequence 194: CP060265.1_strain=SP1451_1
Sequence 195: CP060269.1_strain=SP1380_1
Sequence 196: CP013672.1_strain=AP53_1
Sequence 197: CP014139.1_strain=MEW123_1
Sequence 198: AP014585.1_strain=MTB314_1
Sequence 199: CP010449.1_strain=NGAS322_1
Sequence 200: CP010450.1_strain=NGAS638_1
Sequence 201: NC_011375.1_strain=NZ131_1
Sequence 202: CP012045.1_strain=HKU488_1
Sequence 203: NC_007297.2_strain=MGAS5005_1
Sequence 204: CP011068.1_strain=STAB10015_1
Sequence 205: NC_002737.2_strain=SF370_1
Sequence 206: CP066541.1_strain=MGAS270_1
Sequence 207: NC_004606.1_strain=SSI-1_1
Sequence 208: NC_004070.1_strain=MGAS315_1
Sequence 209: CP072523.1_strain=SHZ-1_1
Sequence 210: CP060639.1_strain=TSPY153_1
Sequence 211: CP060640.1_strain=TSPY764_1
Sequence 212: LR590483.1_strain=NCTC8318_1
Sequence 213: CP035426.1_strain=emm57_1
Sequence 214: CP035428.1_strain=emmSTG866.1_1
Sequence 215: CP035450.1_strain=emm93.4_1
Sequence 216: CP035451.1_strain=emm230_1
Sequence 217: CP032699.1_strain=TSPY155_1
Sequence 218: CP031621.1_strain=MGAS28746_1
Sequence 219: CP033621.1_strain=M75_1
Sequence 220: CP033335.1_strain=TSPY208_1

Sequence 221: CP035430.1_strain=emm55_1
Sequence 222: CP035442.1_strain=emm25_1
Sequence 223: CP035432.1_strain=emm11_1
Sequence 224: CP020082.1_strain=STAB120304_1
Sequence 225: CP020027.1_strain=STAB090229_1
Sequence 226: LR134314.1_strain=NCTC8302_1
Sequence 227: CP029694.1_strain=ABC020055975_1
Sequence 228: LS483382.1_strain=NCTC13738_1
Sequence 229: LS483522.1_strain=NCTC8224_1
Sequence 230: LS483384.1_strain=NCTC13743_1
Sequence 231: LS483421.1_strain=NCTC10877_1
Sequence 232: LS483344.1_strain=NCTC12840_1
Sequence 233: LS483521.1_strain=NCTC8316_1
Sequence 234: LS483351.1_strain=NCTC8195_1
Sequence 235: LS483347.1_strain=NCTC8324_1
Sequence 236: LS483352.1_strain=NCTC12052_1
Sequence 237: LS483326.1_strain=NCTC12045_1
Sequence 238: LS483298.1_strain=NCTC8225_1
Sequence 239: LS483437.1_strain=NCTC13751_1
Sequence 240: CP014542.1_strain=STAB14018_1
Sequence 241: CP015238.2_strain=NS53_1
Sequence 242: NC_017053.1_strain=MGAS1882_1
Sequence 243: NC_017040.1_strain=MGAS15252_1
Sequence 244: CP067090.1_strain=MGAS10870_1
Sequence 245: NC_008022.1_strain=MGAS10270_1
Sequence 246: NC_006086.1_strain=MGAS10394_1
Sequence 247: CP011415.1_strain=D471_1
Sequence 248: CP011414.1_strain=JRS4_1
Sequence 249: CP007240.1_strain=7F7_1
Sequence 250: CP008695.1_strain=M23ND_1
Sequence 251: CP007562.1_strain=NGAS327_1
Sequence 252: CP035433.1_strain=emm65_1
Sequence 253: LR130239.1_strain=PS003_1
Sequence 254: CP033337.1_strain=TSPY453_1
Sequence 255: LS483353.1_strain=NCTC4001_1
Sequence 256: LS483356.1_strain=NCTC8230_1
Sequence 257: LS483386.1_strain=NCTC13742_1

Sequence 258: LS483415.1_strain=NCTC8304_1
Sequence 259: LS483399.1_strain=NCTC8227_1
Sequence 260: LS483338.1_strain=NCTC12064_1
Sequence 261: LS483331.1_strain=NCTC12057_1
Sequence 262: LR134284.1_strain=NCTC8232_1
Sequence 263: LS483391.1_strain=NCTC8320_1
Sequence 264: NC_003485.1_strain=MGAS8232_1
